# Dynamics of the adhesion complex of the human pathogens *Mycoplasma pneumoniae and Mycoplasma genitalium*

**DOI:** 10.1371/journal.ppat.1012973

**Published:** 2025-03-28

**Authors:** David Vizarraga, Akihiro Kawamoto, Marina Marcos-Silva, Jesús Martín, Fumiaki Makino, Tomoko Miyata, Jorge Roel-Touris, Enrique Marcos, Oscar Q. Pich, David Aparicio, Ignacio Fita, Makoto Miyata, Jaume Piñol, Keiichi Namba, Tsuyoshi Kenri

**Affiliations:** 1 Instituto de Biología Molecular de Barcelona (IBMB-CSIC), Parc Científic de Barcelona, Barcelona, Spain; 2 Departament de Química Inorgànica i Orgànica, Universitat de Barcelona, Barcelona, Spain; 3 Institute for Protein Research, Osaka University, Suita, Osaka, Japan; 4 Departament de Bioquímica i de Biologia Molecular and Institut de Biotecnologia i Biomedicina, Universitat Autònoma de Barcelona, Cerdanyola del Vallès, Spain; 5 Graduate School of Frontier Biosciences, Osaka University, Suita, Osaka, Japan; 6 JEOL YOKOGUSHI Research Alliance Laboratories, Osaka University, Suita, Osaka, Japan; 7 JEOL Ltd., Akishima, Tokyo, Japan; 8 Laboratori de Recerca en Microbiologia i Malalties Infeccioses, Hospital Universitari Parc Taulí, Institut d’Investigació i Innovació Parc Taulí (I3PT-CERCA) and Institut de Biotecnologia i Biomedicina, Universitat Autònoma de Barcelona, Sabadell, Spain; 9 Graduate School of Science, Osaka Metropolitan University, Osaka, Japan; 10 Department of Bacteriology II, National Institute of Infectious Diseases, Musashimurayama, Tokyo, Japan; Deutsches Elektronen-Synchrotron, GERMANY

## Abstract

*Mycoplasma pneumoniae* and *Mycoplasma genitalium* are bacterial wall-less human pathogens and the causative agents of respiratory and reproductive tract infections. Infectivity, gliding motility and adhesion of these mycoplasmas to host cells are mediated by orthologous adhesin proteins forming a transmembrane adhesion complex that binds to sialylated oligosaccharides human cell ligands. Here we report the cryo-EM structure of *M. pneumoniae* P1 adhesin bound to the Fab fragment of monoclonal antibody P1/MCA4, which stops gliding and induces detachment of motile cells. The epitope of P1/MCA4 involves residues only from the small C-domain of P1. This epitope is accessible to antibodies only in the “closed conformation” of the adhesion complex and is not accessible in the “open” conformation, when the adhesion complex is ready for attachment to sialylated oligosaccharides. Polyclonal antibodies generated against the large N-domain of P1 or against the whole ectodomain of P40/P90 have little or no effects on adhesion or motility. Moreover, mutations in the highly conserved Engelman motifs found in the transmembrane helix of *M. genitalium* P110 adhesin also alter adhesion and motility. These results show that antibodies directed to the C-domain of P1 hinder the large conformational rearrangements in this domain required to alternate between the “open” and “closed” conformations of the adhesion complex. Since transition between both conformations is essential to complete the attachment/detachment cycle of the adhesion complex, interfering with the gliding of mycoplasma cells and providing a new potential target to confront *M. pneumoniae* and *M. genitalium* infections.

## Author summary

*Mycoplasma pneumoniae* and *Mycoplasma genitalium* are two closely related cell wall-less bacteria and both are relevant human pathogens. *M. pneumoniae* is the primary agent of community-acquired pneumonia and has been implicated in co-infections with other respiratory pathogens. *M. genitalium* infects the reproductive track and is an emerging sexually transmitted “superbug” with a rising prevalence. Our work is mainly focused in how these mycoplasmas interact with the host cells. This is essential both to understand the biology of these microorganisms and for the development of novel therapeutic strategies. These mycoplasmas have reduced genomes but, despite their apparent simplicity, are capable to adhere and to glide on mucosal surfaces and this is critical for virulence and pathogenicity. In this work we show that a mycoplasma protein complex, called Nap, is involved in adhesion and experience large structural transformations during the gliding process. To prove this, we first generated antibodies against the adhesins of the Nap and determined the structure of the Nap in complex with a monoclonal antibody that stops gliding motility, which provides a snapshot of the transition between the “open for attachment” and the “closed for attachment” conformations of the Nap. Importantly, this structural information indicates that the small epitope of the monoclonal antibody is critical for Nap function. Hence, this epitope is a new promising target for vaccine development and to address novel therapies to prevent and treat infections by these pathogens.

## Introduction

*Mycoplasma pneumoniae* and *Mycoplasma genitalium* are closely related human pathogens, sharing a similar genome organization and most of their genes. *M. pneumoniae* is one of the leading microorganisms causing community acquired bacterial pneumonia [1], and coinfections of this mycoplasma with other respiratory pathogens, such as SARS-CoV-2, Adenovirus or *Chlamydia pneumoniae*, have been associated with importantly increased morbidities [1–3]. Recent pneumonia outbreaks in Europe and Asia due to the re-emergence of *M. pneumoniae* also underlines the clinical relevance of this human pathogen [4]. However, the *M. pneumoniae* tropism for respiratory tissues might also be exploited for biomedical applications, and attenuated strains of this mycoplasma have been recently engineered to be used as living pills to treat pulmonary diseases [5]. *M. genitalium* primarily colonizes the urogenital tract but can also infect other tissues such as the rectal and respiratory tracts [6] and has a rising prevalence, particularly in underdeveloped countries, being currently recognized as a sexually transmitted superbug concerning public health [7].

Both mycoplasmas exhibit a membrane protrusion at one of cell poles, referred as the “attachment” or “terminal” organelle (TO), that is instrumental for infection and gliding motility. Cells bind to glass surfaces coated with sialylated oligosaccharides (SOs), which are abundant on the human epithelial surfaces, and glide in the direction of the TO [8–10]. Gliding motility is required to colonize the mucosal surfaces of animal models[11,12] and these mycoplasmas most likely utilize this motile mechanism to span the gel layer mucus barrier before reaching the underlying epithelial target cells [13]. The type of motility of these mycoplasmas is shared only by mycoplasma species belonging to the *M. pneumoniae* cluster [9,14]. The molecular machinery for motility is located in the TO and involves about fifteen different proteins organized as surface structures and internal parts [14–16]. The main and most abundant surface structure is the adhesion complex, also known as the Nap complex, which comprises the transmembrane adhesin proteins P40/P90 and P1 [17–19]. A similar organization of the adhesins P110 and P140 forming a 540-kDa membrane complex is found in *M. genitalium* [16,20]. Adhesins play an essential role in attachment to surfaces by binding to the SOs ligands also allowing mycoplasmas to glide across these surfaces [20,21].

During the last few years, several high-resolution structures have been determined for *M. genitalium* adhesins P140 and P110 [22–24] and *M. pneumoniae* adhesins P1 and P40/P90 [19]. Adhesins of *M. pneumoniae* and *M. genitalium* are orthologous proteins, having a closely related structural organization with sequence identities percentages of 45% between P1 and P140 and of 50% between P40/P90 and P110. Moreover, sequence identities rise above 80% in the transmembrane and cytoplasmic regions [19], with highly conserved Engelman motifs present in the transmembrane helices [22]. Engelman motifs are reported as the most recurrent motif in the association of transmembrane helices [25], frequently providing optimized interfaces for helix-helix interactions [26]. The Nap complexes were proposed to cycle between “open” and “closed” conformations where the SOs binding pockets become accessible or inaccessible, respectively. The SO binding pocket is located in the N-terminal domain of P40/P90 and P110, although accessibility to the pocket is determined by the interaction with the N-terminal domain of P1 and P140 that is tighter in the “closed” conformation. Adhesins P1 and P40/P90, together with protein P116, are the most immunogenic proteins of *M. pneumoniae* [19,27]. To study the immunogenic and functional properties of P1, monoclonal antibodies were generated against a fragment of this adhesin spanning from residue Ala1160 to Gln1518, (P1:1160-1518 peptide). One of the monoclonal antibodies obtained (henceforth referred as P1/MCA3) was reported some years ago to progressively reduce gliding speed, removing mycoplasma cells from surfaces in a concentration-dependent way, while having little effect on non-moving cells [28]. These observations led to the conclusion that during gliding, P1 has to experience important conformational changes, participating like a leg in a “power stroke” that propels the cell, with epitope exposure to antibody P1/MCA3 significantly reduced in non-moving cells.

How the functioning of the Nap complex is related to the motility of mycoplasma cells remains elusive. Monoclonal antibody P1/MCA3 is no longer available and its amino acid sequences remain unknown. However, the monoclonal antibody P1/MCA4, which was also obtained by immunizing with the same antigen and in parallel to P1/MCA3, is still available and shows comparable effects on the adhesion and motility of *M. pneumoniae* cells (S1 Table). We have now solved by cryo-electron microscopy (cryo-EM) the structure of P1 interacting with the Fab fragment of P1/MCA4. The structure of the P1-Fab(P1/MCA4) complex provides a snapshot of the “closed” conformation of P1. We have also determined how motility is affected by polyclonal antibodies against the whole ectodomains of adhesins P1 and P40/P90 or against constructs of the N-terminal domain of P1. To investigate how the observed conformational changes experienced by the ectodomains relate with the transmembrane and intracellular regions of adhesins, we have mutated the three highly conserved Engelman motifs (GXXXG) [25] found in the transmembrane helices of adhesins. Altogether, these results explain the specific neutralization mechanism deployed by antibodies against the C-domain of P1 and provide a deep insight into the functioning of the Nap complexC-domain.

## Results

### Monoclonal antibody P1/MCA4 and its binding to P1

Monoclonal antibody P1/MCA4, raised against the P1:1160-1518 peptide, strongly inhibited the adhesion of *M. pneumoniae* to red blood cells*.* In the presence of P1/MCA4, gliding mycoplasma cells rapidly reduce their speed and eventually halt and detach from sialylated glass surfaces (S1 Video), while non-moving cells are not affected by the presence of P1/MCA4 and remain attached to surfaces. As mentioned in the introduction, a similar behaviour had been reported for antibody P1/MCA3 [28]. It is worth mentioning that this behaviour is also observed in three additional monoclonal antibodies raised in the same batch as P1/MCA3 and P1/MCA4 (S1 Table). The P1:1160-1518 peptide corresponds to a fragment of the N-terminal domain (residues Ala1160-Thr1399) and to (almost) the whole C-domain (Ala1400-Gln1518) according to the structural information now available [19].

cDNA sequencing of P1/MCA4 mRNAs in hybridoma cells showed the presence of a single IgG heavy chain mRNA but two different light chain mRNAs (S1 Fig). N-terminus analysis of samples from antibody P1/MCA4 (obtained as indicated in the Material and Methods) confirmed the presence of one heavy chain but two different light chains (roughly 70% for the most abundant). The heterogeneity of light chains might explain the failure of numerous crystallization attempts involving P1/MCA4. Analysis by multi-angle light scattering (MALS) showed the formation of a stable and homogeneous P1-Fab(P1/MCA4) complex, in a solution with equimolecular amounts of P1/MCA4 Fabs and the whole P1ectodomain (residues Thr29-Asp1521), indicating that the two kinds of Fab found in P1/MCA4 bind efficiently to P1 (S2 Fig).

Antibody P1/MCA4 presents high affinity for a construct corresponding to the C-domain of P1, proving that this small domain contains the epitope. Moreover, western blotting analysis performed using different segments of P1 showed that residues Thr1426-Asp1438 are a key part of the epitope (S3 Fig). An additional observation that could have clinical implications is that P1/MCA4 presents affinity also for the C-domain of P140 (residues Lys1220-Asp1351) from *M. genitalium,* even stopping the movement of cells, although after treatments longer than with *M. pneumoniae*, in agreement with the high sequence and structural similarities between the C-domains of the adhesins of *M. pneumoniae* and *M. genitalium* (S4 Fig).

### Cryo-EM structure of the P1-Fab (P1/MCA4) complex

**Fig 1:**
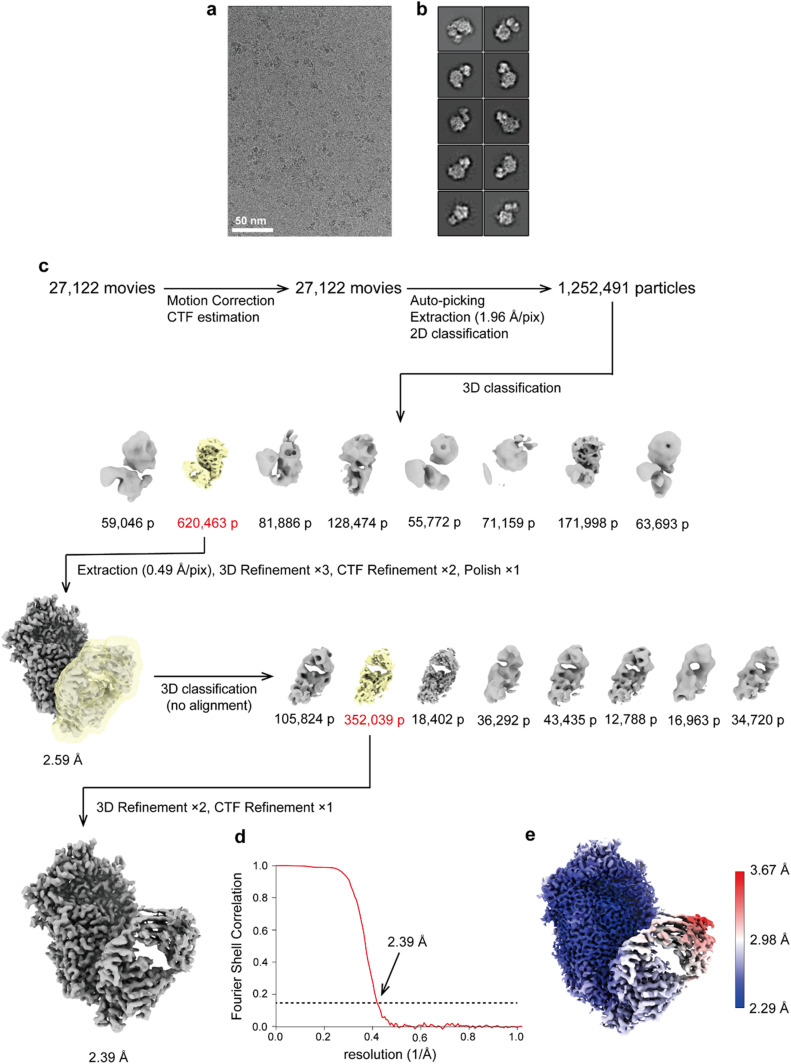
Cryo-EM structure determination of the P1-Fab(P1/MCA4) complex. a) Representative micrograph of P1-Fab(P1/MCA4) complexes examined by CryoEM. b) Initial classes obtained after 2D classification of 2000 particles that were manually selected. c) Schematic processing overview for the P1-Fab(P1/MCA4) complex structure resolution from particles that were automatically picked from movies; CTF, Constrast transfer function. d) Fourier shell correlation of P1-Fab(P1/MCA4) complex at a final resolution of 2.39Å according to the 0.143 cut-off criteria. e) Final 3D reconstruction map color-coded according to local resolution.

**Fig 2:**
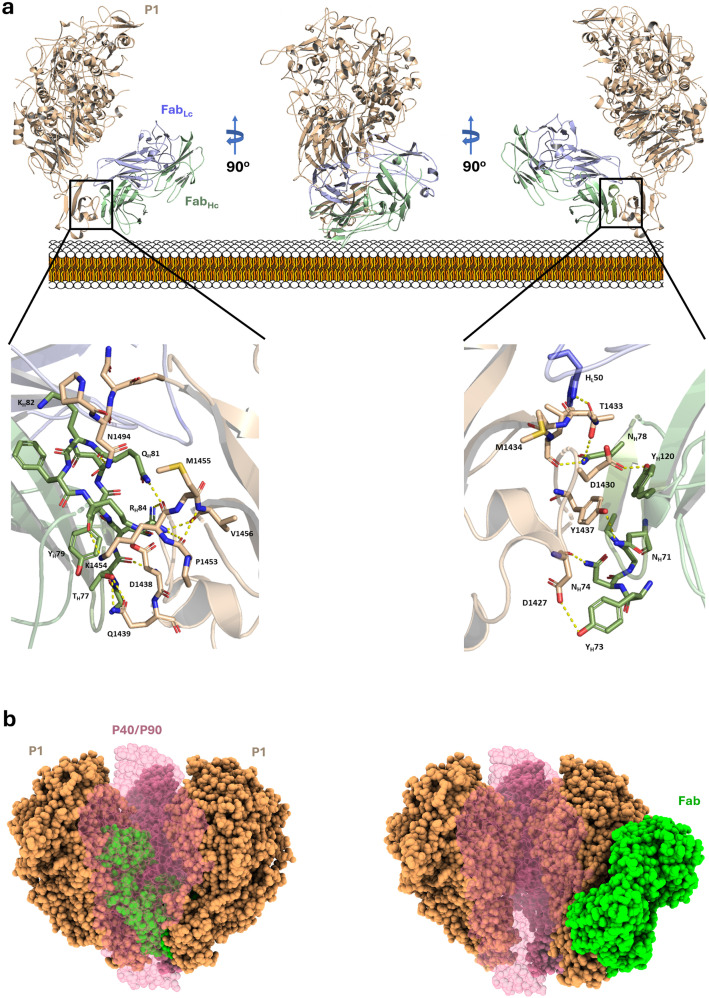
Cryo-EM structure of the P1-Fab(P1/MCA4) complex. a) Ribbon representations, with three 90° apart views, of the complex between the ectodomain of P1 (brown) and the Fab fragment of Mab P1/MCA4 (light and heavy chains in blue and green, respectively). Insets indicate the main interactions between P1 and the Fab. The epitope is located in the C-domain of P1 closed to the mycoplasma membrane, which is also displayed as a reference. b) Two views of the Fab(P1/MCA4) (shown in green) and the extracellular region of the Nap, with P1 (brown) and P40/P90 (pink transparent). In the “open” conformation of the Nap (left panel) the P1/MCA4 epitope, buried at the center of the Nap, is totally inaccessible to antibodies and a Fab placed there would catastrophically clash with the adhesins. Therefore, to expose the P1/MCA4 epitope the C-domain of P1 has to experience an important rearrangement (right panel).

The structure of the ectodomain of P1 bound to the Fab fragment of P1/MCA4 was determined by cryo-EM single particle analysis with an overall resolution in the final map of 2.4 Å (Figs 1 and S5 and S2 Table). The high quality of the map allowed accurate modelling of the structure from P1 and the Fab variable domains, defining clearly the paratope-epitope interface (Fig 2a). The Fab constant domains were less well defined, indicating some flexibility in the Fab elbow. The sequence observed for the Fab corresponded to the one with light chain L1. The structure of P1 consists of a large N-terminal domain (residues 60-1399) and a smaller C-domain (1400-1521), in full agreement with the structures of P1 reported previously [19]. Superposing the P1 structure determined here with the P1 crystal structure (PDB code 6rc9), gives a root mean square deviation (rmsd) of 1.80 Å for 1236 aligned residues (from a total of 1339 in the model) (S6 Fig). When the superposition is done separately for the N-terminal domain and C-domain, rmsds are 1.65Å (1151 aligned residues out of 1226) and 1.20Å (97 aligned out of 104), respectively. These results show the high plasticity of P1, in particular for the N-terminal domain, which experiences significant changes although it participates only indirectly in the interaction with the P1/MCA4 Fab. Comparison of the crystal (6rc9) and the new (in the complex of P1-Fab) structures of P1, indicates a 7.7° rotation of the C-domain relative to the N-terminal domain, confirming hinge movements between the two domains as had suggested the cryo-EM structure of P1 where the C-domain was disordered [19]. The structure of the P1-Fab complex shows that the epitope of P1/MCA4 is conformational, although involving residues from the C-domain only, in particular from loop Val1425-Asp1438 in agreement with the epitope mapping analysis (Figs 2a and S3). The quality of the cryo-EM map was high enough to even allow the localization in the N-terminal domain of a significant number of solvent molecules, most of them also present in the crystal structure of P1 (S5 Fig). It is worth mentioning that docking using a previous cryo-EM map determined at lower resolution, correctly predicted the overall binding interface between P1 and the Fab(P1/MCA4), with the largest deviations arising from the inaccuracy on the fitting of the P1 C-domain (S7 Fig).

Importantly, superposing the structure of P1-Fab(P1/MCA4) onto the Nap complex in “open” conformation available for *M. genitalium* [22] results in a catastrophic steric clash of the Fab against the adhesins, indicating that the epitope of P1/MCA4 is totally inaccessible to antibodies in the “open” conformation of the Nap complex (Fig 2b). Therefore, this complex must experience important structural rearrangements between the “open” conformation and when the epitope of P1/MCA4 is fully exposed.

### Polyclonal antibodies against the P40/P90 and P1 ectodomains and against the N-terminal domain of P1

Polyclonal antisera against the ectodomains of P40/P90 (residues Ala25-Pro1113) and of P1(residues Thr29-Asp1521) were obtained by immunizing mice with recombinant versions of these polypeptides. Polyclonal antisera against the N-terminal domain of P1 were obtained by immunizing mice with the P1 N-terminal domain (residues Thr29-Ala1375). These antisera were tested by ELISA, Western Blotting and immunofluorescence assays on whole *M. pneumoniae* cells, and titers higher than 1/2000 were obtained for all the antisera. Effects of these antisera on *M. pneumoniae* motility and attachment were investigated by incubating *M. pneumoniae* cells in the presence of 3% gelatin in the SP4 medium [21]. Because *M. pneumoniae* cells spontaneously detach at high frequencies from the observation surface in the absence of gelatin [27], more reproducible and consistent results were obtained when gliding motility was analyzed in SP4 medium supplemented with gelatin (Tables 1 and S3). In the absence of antibodies (negative control), most of the cells remained attached and motile during the observation time (Table 1). In contrast, the P1 ectodomain antisera stopped the movement of 50% of mycoplasma cells after four minutes of incubation (Table 1 and S2 Video) with very few cells remaining motile at the end of the observation period. No significant effects on mycoplasma gliding were detected neither for the antisera against the P1 N-terminal domain nor for the P40/P90 ectodomain, even at the lowest dilution tested (Table 1 and S3 and S4 Videos). It is also worth mentioning that cell adhesion was inhibited when cells were added to media that already contained antisera against the ectodomains of either P1 or P40/P90, suggesting that these antibodies can interfere with their binding to SOs via steric hindrance, but their epitopes are not accessible when the cells are attached to surfaces.

**Table 1:** Mycoplasma motility in the presence of antibodies.

Antibody or sera	SP4				SP4 3% gelatin		
**Before** ^#^	**After** ^#^			**Before** ^#^	**After** ^#^	
**Motile cells (% ± SE)**	**Motile cells (% ± SE)**	**RT**_**50**_ **(min ± SE)**^&^	**Detached cells (% ± SE)**	**Motile cells (% ± SE)**	**Motile cells (% ± SE)**	**RT**_**50**_ **(min ± SE)**^&^
**Control** ^$^	89.9 ± 1.2	76.1 ± 4.8	NA	51.5 ± 4.3	96.3 ± 0.3	96.5 ± 0.1	NA
**P1**	89.4 ± 0.6	1.8 ± 1.8	4.65 ± 1.2	72.3 ± 2.4	96.5 ± 0.5	3.4 ± 2.8	4.1 ± 0.35
**P40/P90**	87.5 ± 3.9	74.3 ± 4.4	NA	45.2 ± 26.1	96.4 ± 0.4	95.7 ± 0.3	NA
**P1 N-ter**	95.6 ± 0.1	85.4 ± 1.6	NA	59.8 ± 0.1	88.9 ± 3.2	88.2 ± 2.7	NA
**P1/MCA4**	–	–	–	–	97.3 ± 0.7	0 ± 0	2.6 ± 0.26

$ Negative control. PBS with no antibody was added.

# Measurements made “before” or “after” adding antibodies or a control PBS solution. (NA): Not applicable. (–): Not performed. SE: standard error.

& RT_50_ is the antibody incubation time required to stop the movement of 50% of motile cells.

Binding of antibodies to epitopes whose accessibility varies during the conformational transformation interferes with the dynamics of the adhesion complex. Accordingly, the attachment/detachment cycle is halted by antibody P1/MCA4 against the C-domain of P1 that experiences important rearrangements during the cycle. In contrast, the attachment/detachment cycle is not altered, or very little, by antibodies against P40/P90 or against the N-terminal domain of P1, indicating that accessibility of their epitopes must remain essentially unchanged during the cycle.

### Mutations in the Engelman motifs

**Fig 3:**
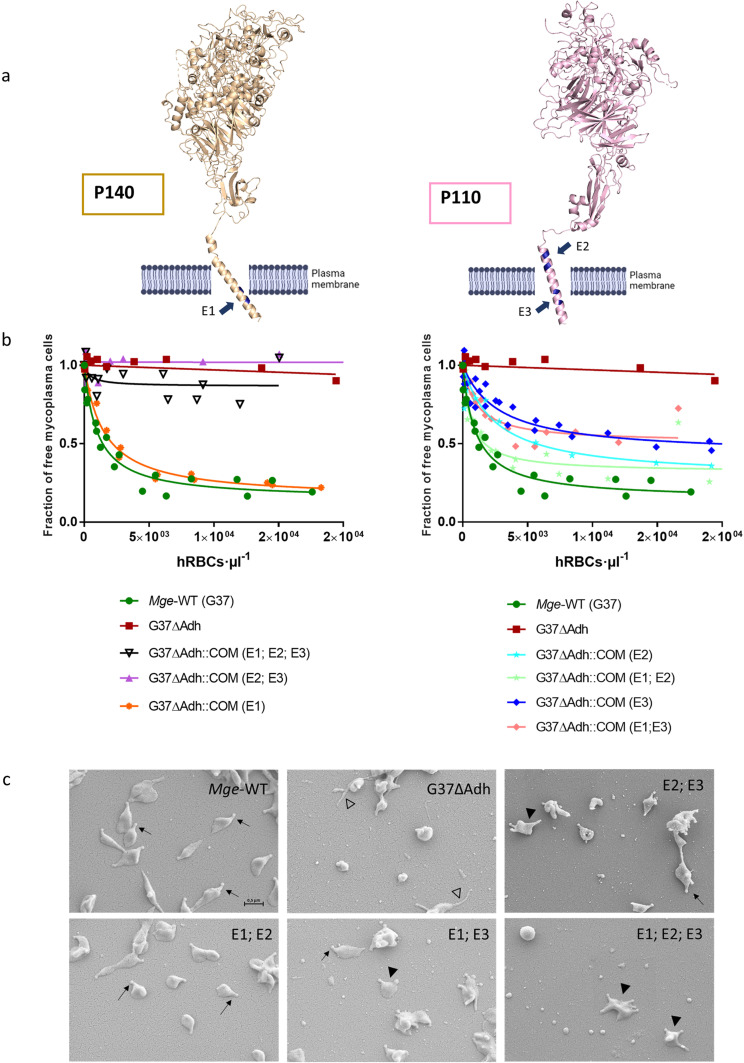
Engelman motifs in adhesins. a) Transmembrane helices of adhesins from the *M. pneumoniae* cluster of mycoplasmas contain three highly conserved Engelman motifs (GxxxG sequences), referred as E1 (P140) and E2, E3 (P110) in *M. genitalium*. b) Mutational analysis, performed in the Engelman motifs of P140 and P110, indicate that motifs E2 and E3 from P110 have important and synergic contributions to adhesion. On the contrary, no effects were observed when E1 from P140 was mutated. c) Changes in adhesion correlate with increasing rates of cells presenting multiple TOs phenotypes.

The transmembrane domains of P1 and P40/P90, predicted to be single helices, are located just after the C-terminal of the globular ectodomains of these proteins. Remarkably, these helices contain one Engelman motif in P1 and two Engelman motifs in P40/P90 (S8 Fig). These three motifs are highly conserved in the *M. pneumoniae* cluster of mycoplasmas. Engelman motifs are frequently involved in high-affinity interactions between membrane helices and have the sequence GXXXG, with X being any residue but in general hydrophobic [25]. We have investigated by mutational analysis possible contributions of the Engelman motifs from adhesins to the organization and functioning of the Nap complex. This study was performed with *M. genitalium* because simple genetic methods to introduce point mutations are available in this microorganism and transmembrane helices from orthologue adhesins of *M. pneumoniae* and *M. genitalium* present high sequence identities (S8 Fig). The Engelman motif at the C-end of the P140 transmembrane helix, close to the internal side of the cell membrane, will be referred as E1 and the two Engelman motifs in P110 close to the external and to the internal sides of the cell membrane, as E2 and E3, respectively (Fig 3a). Mutations were introduced by transposon delivery in a *M. genitalium* null mutant G37∆Adh (G37∆MG_191-∆MG_192), to obtain isogenic strains bearing Gly to Phe double point mutations of the glycine residues of P140 and/or P110 Engelman motifs (S4 Table). The protein profile analysis of these mutant strains showed that adhesins bearing the different mutations were expressed at similar levels that P140 and P110 in wild type (WT) cells (S9 Fig). The properties of the adhesin variants were tested by a quantitative hemadsorption assay (Fig 3b). This assay provides data about the adhesion properties of mycoplasma cells to mammalian red blood cells [29]. Cells from E1 mutant strain showed hemadsorption values very similar to WT cells and also similar to the cells from the null mutant G37∆Adh complemented by a WT copy of both adhesin coding genes. In contrast, E2 mutants showed an intermediate adherent phenotype and E3 mutants had a more severely impaired hemadsorption phenotype, characterized by low B_max_ and high Kd values, indicating the presence of adhesins with a lower binding affinity for cell receptors. As expected, E1-E2 and E1-E3 mutant strains exhibited phenotypes similar to those showed by E2 and E3 mutants, respectively. In addition, E2-E3 and E1-E2-E3 mutant strains exhibited a non-adherent phenotype characterized by very low B_max_ and very high K_d_ values, resembling the phenotype of the cells from the G37∆Adh null mutant strain. Interestingly, immunolabeling with a polyclonal antiserum against the Nap complex indicates that adhesin variants are located at the tip of the cells bearing the triple mutation E1-E2-E3 (S10 Fig), suggesting the presence of Nap complexes in TOs of these cells. Mutant strains were also examined by Scanning Electron Microscopy (Fig 3c). While cells from E1 mutants exhibited normal morphologies, cells with mutations in E2 or E3 showed a multiple TO (mTO) phenotype. Although the fraction of cells presenting mTOs was only slightly increased in E2 variants, most of the cells from E2-E3 and E1-E2-E3 variants exhibited mTOs (S5 Table). Nascent TOs develop adjacent to a preexistent TO, and gliding motility provides the driving force to deliver the new TOs to the opposite cell pole. This process is hindered in cells having adhesion and motility deficiencies [30], which can explain the correlation between the reduced adhesion and the increased frequency of cells showing mTOs in E2-E3 variants. Since P110 and P140 adhesins co-stabilize each other and the formation of Nap complexes is essential for TO development [31,32], the presence of TOs by SEM suggests that these adhesins are properly folded in the TOs of cells bearing Engelman motif variants. Moreover, the absence of detectable phenotypic defects in E1 adhesin variant also indicates that this mutation has no impact in the folding or in the organization of the Nap complex. Therefore, mutational analysis allows us to conclude that Engelman motifs from P110, but unexpectedly not from P140, have a critical and synergic contribution to adhesion, strongly suggesting that interactions between the transmembrane helices of the two P110 subunits are required to achieve a functional “open”, ready for binding, conformation of the Nap complex.

## Discussion

The *M. pneumoniae* cluster of mycoplasmas has developed a unique, strikingly complex, molecular machinery for gliding motility that consists of internal and surface structures located in the TO, a morphologically conspicuous cell protrusion. A gliding model has been proposed based mainly on biophysical and structural data of the internal structures [ 9,14,15]. The model is a variant of the “inchworm” model, in which repeated contractions and extensions, synchronized with alternative attachment and detachment of the TO front and rear sides, enable the smooth gliding of cells [8,32,33]. According to this model, adhesins P1 and P40/P90 forming the Nap complex, which is the main surface structure of the TO, have a key contribution to both attachment and movement, by going throughout an iterative four stages cycle during gliding [16]. In the last few years a wealth of high resolution structural data has been obtained for the Nap complex [16,20].

**Fig 4:**
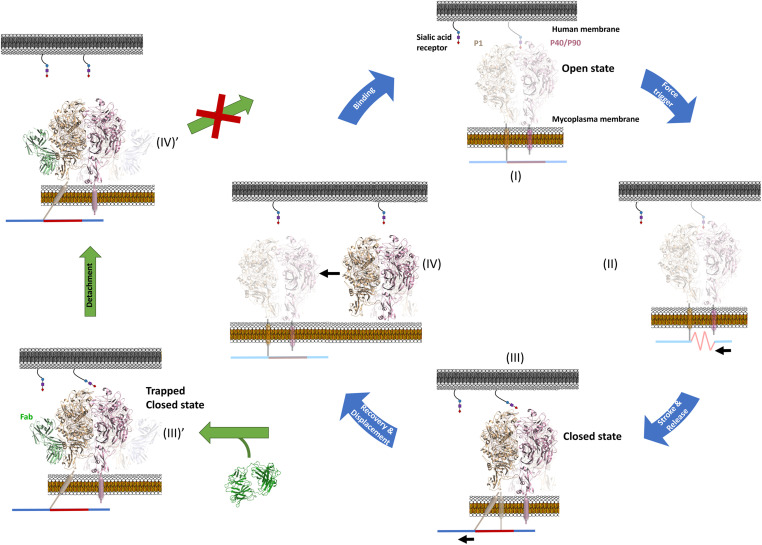
The attachment and detachment cycle of the Nap complex. An active Nap complex cycles between “open” and “closed” states, with the binding site to sialic oligosaccharides becoming alternatively accessible and inaccessible for binding. As shown in this work, during the nap cycle, structural rearrangements are experienced mainly by the C-domain of P1 (brown), with hinge rotations of about 175°, while the ectodomains of P40/P90 (pink) remain mostly unchanged. The wide movement of the C-domain from P1 drags, by contiguity, transmembrane helices thus reaching the internal parts of the TO (represented here simply as a line of elastic (red) and rigid (blue) components with possible displacements shown as black arrows). Binding of antibody P1/MCA (green) to the C-domain of P1 halts the cycle, trapping the “closed” conformation of the Nap. The structures known prior to this work are indicated using soft colors.

The structure of the complex of *M. pneumoniae* adhesin P1 and the Fab fragment from the monoclonal antibody P1/MCA4, determined in this work, shows that the P1/MCA4 epitope involves residues of the C-domain of P1 only (Fig 2a). This epitope is totally inaccessible to antibodies in the “open” (ready for binding to SOs) conformation of the Nap complex (Fig 2b)*.* Therefore, for the P1/MCA4 epitope to be exposed, the adhesion complex has to experience a major rearrangement with respect to the “open” conformation. The conformation where P1/MCA4 binds must be unsuitable for attachment to SOs, as binding of antibody P1/MCA4 slows and induces detachment of moving *M. pneumoniae* cells only. Together with the available information, our results allow now to model accurately the four stages of the Nap complex cycle (Fig 4 and S5 Video) explaining how internal and surface structures interact with each other.

Stage I: The cycle starts with the “open” conformation that is ready for binding to the SOs. The extracellular region from the “open” conformation of the Nap complex was built according to the structure determined *in situ* by cryo-electron tomography (cryo-ET) for *M. genitalium* [22]. The Engelman motifs found in the transmembrane helix of P40/P90 have a critical role to stabilize the “open” conformation, suggesting that the helices from the two P40/P90 subunits interact with each other.

Stage II: Binding to SOs triggers the transition of the Nap complex towards the “closed” conformation. During the transition the exposed surfaces of the P40/P90 subunits remain unchanged, in agreement with the restraints imposed by the interactions between the transmembrane helices of the two P40/P90 subunits and previous studies showing that the two SOs binding sites of the Nap complexes are probably involved [34–36]. In turn, the C-domain of P1 has to experience an important rearrangement to allow the exposure of the P1/MCA4 epitope (S11 Fig).

Stage III: Reaching the “closed” conformation of the Nap complex, where the N-terminal domains of P1 and P40/P90 interact tightly with each other, forcing the release of SOs and occluding the binding site. At this stage the C-domain of P1 has completed a hinge rotation of about 175º with respect to the “open” conformation, allowing the epitope of antibody P1/MCA4 to be fully exposed. Hinge movements of the C-domain must be associated with displacements or distortions of the transmembrane helix, contiguous along the P1 sequence, which provides a way to communicate back and forth structural information between extra- and intra-cellular regions.

Binding of antibody P1/MCA4 to the C-domain of P1 traps the Nap complex in the “closed” conformation, which explains why only moving cells are affected by the antibody (left images in Fig 4). The increasing number of complexes in the closed, non-adherent conformation slows down the speed of gliding and weakens cell attachment.

Stage IV: Recovering the “open” conformation of the Nap complex. The “open” and “closed” conformations of the unbound adhesion complexes as described here so far, have the same molecular components. Assuming no other elements are at play, the least stable conformation must revert spontaneously towards the most stable, while the transition from the most towards the least stable will require energy. The “open” conformation, the most abundant in an *in situ* cryo-ET analysis [22], is likely the most stable, suggesting that the energy input is required for the “open” to “closed” transition associated with the power stroke and the straining of the internal structures (from Stage I to Stage III in **Fig 4**) [16].

Results provide now a consistent structural framework for the functioning of the Nap complex during the attachment/detachment cycle in which subunit P1, mainly its C-domain, experiences extensive rearrangements pivoting around static P40/P90 subunits anchored by interactions of their Engelman motifs. Results also lay out a clear structural explanation for the efficient neutralization mechanism of antibodies such as P1/MCA4, binding to the dynamic and highly conserved C-domain of P1. This antibody shows that gliding motility can be effectively blocked by interfering with the attachment/detachment cycle of the Nap complex, providing a new potential target to confront *M. pneumoniae* and *M. genitalium* infections.

## Materials and methods

### Ethics statement

The experimental procedures to immunize mice and obtaining polyclonal sera were approved by the Ethics Committee on Animal and Human Experimentation of the Universitat Autònoma de Barcelona (UAB, procedure 1002R3R2R). Experiments growing and transforming mycoplasma cells were approved by the Biosecurity Committee of the UAB (procedure HR-640-20).

### Bacterial strains and tissue culture cells

*M. pneumoniae* M129 strain was grown in cell culture flasks containing SP4 or PPLO media and incubated at 37 ºC with or without 5% CO_2_. Surface-attached mycoplasmas were harvested using a cell scraper and resuspended in medium or suitable buffers for experiments. When growing mycoplasma cells on IBIDI 8-well chamber slides, each well was seeded with about 10^5^ CFUs and incubated for 12-24 h in 200 μL SP4 supplemented with 3% gelatin. All *M. genitalium* strains were grown in SP-4 broth at 37°C in a 5% CO_2_ atmosphere in tissue culture flasks. SP-4 plates were prepared supplementing the medium with 0.8% agar (BD). Chloramphenicol (17 μg mL^−1^), puromycin (3 μg mL^−1^) or gentamycin (100 μg mL^−1^) were added for mutant selection.

*Escherichia coli* strain XL-1 Blue (Agilent, Santa Clara, USA) was used for cloning and plasmid propagation purposes. The strain was grown in Luria Bertani (LB) or LB agar plates containing 100 μg mL^−1^ ampicillin, 40 μg mL^−1^ X-Gal, and 24 μg mL^−1^ Isopropyl β-D-1-thiogalactopyranoside (IPTG) when needed.

NSI myeloma cells [37] were grown in RPMI 1640 medium supplemented with 10% FBS and 50 μg mL^−1^ gentamycin (complete RPMI). Hybridomas were selected in complete RPMI supplemented with HAT media and BM-Condimed (Sigma Aldrich).

### Cloning, expression and purification of P1 and P1 fragments

The coding sequences for constructs of P1 and the C-domain of P1 were amplified from the synthetic clone of MPN_141 (P1) gene from *M. pneumoniae* (Genscript), using primers P1F and P1R for P1 (29-1521), P1Ct1400_F and P1R for P1 C-terminal (1400-1521), P1Ct1376_F and P1R for P1 C-terminal (1376-1521) (S6 Table). The coding sequences for constructs of P40/P90 were amplified from the synthetic clone of MPN_142 (P40/P90) gene from *M. pneumoniae* (Genscript), using primers P40P90_F and P40P90_R (23-1114). The PCR fragments were cloned into the expression vector pOPINE (-) to add a C-terminal His-tag to the resulting constructs. Recombinant proteins were obtained after expression with B834 (DE3) cells (Merck), with IPTG at 0.8 mM and 22ºC overnight. The cell pellets were lysed in 1xPBS at pH 7.4 and 40 mM Imidazole (binding buffer) and centrifuged at 20000 RPM at 4ºC. Supernatant was charged into a HisTrap 5ml column (GE Healthcare) and eluted with a buffer containing 400 mM imidazole. Soluble aliquots were concentrated and loaded in a Superdex 200 GL 10/300 column (GE Healthcare) with a Tris·HCl 20 mM buffer (pH 7.4) and 150 mM NaCl.

### Production and validation of polyclonal antibodies

Polyclonal antisera anti-P1, anti-P1 fragments and anti-P90/P40 were prepared by immunizing BALB/C mice with the respective recombinant proteins and polypeptides described above. Sera were obtained by cardiac puncture of properly euthanized mice just before splenectomy and tittered using serial dilutions of each antigen. Titers of the different polyclonal sera were determined as the IC_50_ value from four parameter logistic plots and found to be approximately between 1/2500 and 1/4000.

### Production and sequencing of the monoclonal antibody P1/MCA4

A *p1* gene fragment was amplified by PCR from genomic DNA of *M. pneumoniae* M129 strain using primers P1F_2 and P1R_2 (S6 and S7 Tables). The amplified fragment was inserted into *Nco*I and *Xho*I site of pET-30c(+) expression vector, resulting the pP1-8 plasmid. *E. coli* BL21(DE3) was transformed by the pP1-8 for expression of a recombinant P1 peptide containing Ala1160 to Gln1518 of the P1 of M129 strain. The recombinant P1 was purified by His-tag affinity chromatography and used to immunize mice for production of anti-P1 monoclonal antibody. The monoclonal antibody P1/MCA4 was selected by ELISA screening and showed high specificity to P1 in Western blotting analysis and immunofluorescence microscopy of living *M. pneumoniae* cells. The amino acid sequence of P1/MCA4 was determined at GenScript (Tokyo, Japan) by cloning and sequencing of the cDNAs of antibody mRNA from P1/MCA4 producing hybridoma cell. The obtained sequences for a single H chain and two L chain of the P1/MCA4 (S1 Fig) were deposited into the DDBJ/ENA/GenBank databases under the accession numbers LC600310, LC600311 and LC600312.

### Purification of the fab fragment

Monoclonal antibody P1/MCA4 against P1 was diluted up to 2 mg mL^−1^ in 1xPBS buffered at pH 8.0 and mixed with 0.1mg/ml of Papain, 20 mM L-cysteine, 20 mM EDTA and 1xPBS pH: 7.0 in a relation 1:40 (Protein:Papain) and incubated during 4 hours at 37ºC. To stop digestion, 10% of the final volume of iodoacetamide was added. The digested solution was loaded onto HiTrap Protein G 5ml with Binding buffer (20 Mm Na_2_HPO_4_ pH:7.0) and Elution buffer (0.1 M glycine·HCl pH 2.7). Soluble aliquots were concentrated and loaded in a Superdex 75 GL 10/300 column (GE Healthcare) with a Tris·HCl 20 mM pH 7.4 and 150 mM NaCl buffer. Complexes of P1-mAb and P1-Fab were formed by mixing both proteins in a ratio 2:1 and 1:1, respectively.

### Epitope mapping of monoclonal antibody P1/MCA4

For epitope mapping of P1/MCA4, a series of plasmids that express recombinant P1 peptides were constructed (S7 Table). These plasmids were generated by partial deletion of the pP1-8 plasmid using specific primer sets and PrimeSTAR Mutagenesis Basal kit (Takara Bio, Shiga, Japan). Recombinant P1 peptides were produced in *E. coli* BL21(DE3) harboring the plasmids, separated by 10-20% gradient-gel SDS-PAGE, and transferred to nitrocellulose membranes. Recombinant P1 peptides on membranes were reacted with 0.5 µg/ml P1/MCA4. After washing of the membranes, binding of the P1/MCA4 to P1 peptide was detected by anti-mouse IgG, HRP conjugate secondary antibody (Promega, Madison, WI, USA) and EzWestBlue W substrate (Atto, Tokyo, Japan)*.*

### Size exclusion chromatography and multi angle light scattering (SEC-MALS)

Molecular weights were measured using a Superdex 200 10/300 GL (GE HEalthcare) column in a Prominence liquid chromatography system (Shimadzu) connected to a DAWN HELEOS II multi-angle light scattering (MALS) detector and an Optilab T-REX refractive index (dRI) detector (Wyatt Technology). ASTRA 7 software (Wyatt Technology) was used for data processing and result analysis. A dn/dc value of 0.185 mL g^−1^ (typical of proteins) was assumed for calculations.

### Single-particle cryo-EM of the binary complex

To prevent preferred orientations, Octylthioglucoside (OG) was added to solution of purified P1-Fab (P1/MCA4) complex to adjust the final concentration of OG to 0.9% (w/l). 2.6 μL of sample solution was applied to a glow-discharged Au-coated Quantifoil holey carbon grid (R1.2/1.3, Cu, 200 mesh), blotted for 4 sec at 4 °C in 100% humidity and plunged into frozen liquid ethane using a Vitrobot Mark IV (Thermo Fisher Scientific). The grid was inserted into a CRYO ARM 300 (JEOL) operating at an acceleration voltage of 300 kV. CryoEM images were recorded with a K3 direct electron detector (Gatan) in CDS mode with an energy filter at a slit width of 20 eV. Data were automatically collected using the SerialEM software (https://bio3d.colorado.edu/SerialEM/) at a physical pixel size of 0.49 Å with 50 frames at a dose of 2.0 e^−^/ Å ^2^ per frame and an exposure time of 2.86 sec per movie with a defocus ranging from -0.5 to -1.5 μm. A total of 27,122 movies were collected for P1-Fab complex.

The movie frames were subjected to beam-induced movement correction using MotionCor2.1 [38] and contrast transfer function (CTF) was evaluated using Gctf [39]. Approximately 2,000 particles were manually selected from 20 micrographs to perform two-dimensional (2D) classification. Using a good 2D class average image, a total of 4,312,408 particle images were automatically picked and 2D classifications were performed using RELION-3.1 [40]. A total of 1,252,491 particles were selected for building the initial model of the P1-Fab complex using cryoSPARC2 [41] and subjected to 3D classification into 8 classes using RELION-3.1. The selected particles were re-extracted at a pixel size of 0.49 Å and subjected to three 3D refinement, two CTF refinement and Bayesian polishing. After selection of particles using no-alignment 3D classification, a total of 352,039 particles were subjected to two 3D refinement and CTF refinement. The final 3D refinement and post-processing yielded maps with global resolutions of 2.39 Å, according to the 0.143 criterion of the Fourier shell correlation (S2 Table). Local resolution was estimated using RELION-3.1. Processing strategy is described in Fig 1.

### Model building and refinement of the binary complex

The model of the P1-Fab(P1/MCA4) complex was built based on the cryoEM density map. Previous published crystal structure of P1 (PDB ID: 6RC9) was docked into the EM density map using UCSF Chimera [41]. N-domain, C-domain of P1 and Fab were manually repositioned and refined iteratively using COOT [42] and PHENIX real space refinement [43]. The statistics of the 3D reconstruction and model refinement are summarized in S2 Table. The final refined structure has been deposited in the PDB with code 8ROR.

### DNA manipulation and primers

Plasmid DNA was purified using GeneJET Plasmid Miniprep Kit (Thermo Fisher Scientific). PCR products and DNA fragments were recovered from agarose gels using NucleoSpin Gel and PCR Clean-up Kit (Macherey-Nagel, Düren, Germany), and digested using the corresponding restriction enzymes (Thermo Fisher Scientific) when necessary. For transformation of *M. genitalium*, plasmids were purified using the GenElute HP Midiprep Kit (Sigma-Aldrich, St. Louis, USA) following the manufacturer’s instructions. All primers used in this study are listed in S8 Table.

### *M.* *genitalium* mutant strains

The suicide plasmid pBE∆MG_191/MG_192 was designed to generate a G37 *M. genitalium* MG_191 and MG_192 null mutant strain by gene replacement. First, a 1 kb flanking upstream region (UR) to the MG_191 gene was PCR-amplified using the MgParUp-F and MgParUp-R primers. Similarly, a 1 kb flanking downstream region to the MG_192 gene was PCR-amplified with the MgParDw-F and MgParDw-R primers. In parallel, the a lox version of the CmM438 selectable marker [44] was PCR-amplified with the Lox71p438-Fwd-XhoI and CatLox66-Rev-BamHI primers, obtaining the CmM438 flanked with the lox61 and lox71 sequences. Then, the UR and DR PCR products were joined by PCR with the MgParUp-F and MgParDw-R primers. The resulting PCR product was cloned into an EcoRV-digested pBE plasmid [45]. Finally, the resulting CmM438 amplicon was digested with XhoI and BamHI restriction enzymes and ligated into the similarly digested pBE containing the UR and DR regions obtained before.

This plasmid was used to obtain the G37∆Adh chloramphenicol resistant mutant strain. Electroporation of this mutant with the pCre plasmid [46] that contains the Cre recombinase allowed us to obtain the G37∆Adh strain, free of any antibiotic selectable marker. This strain was used as the recipient strain to transform all the pMTnPac plasmids generated in this study.

MG_191 and MG_192 genes from the chromosome of *M. genitalium* G37 strain were amplified by PCR using COMmg191-F-ApaI and COMmg191/192-R-SalI primers. The resulting PCR product was cloned into an EcoRV-digested pBE plasmid to create the pBE-COMP140P110. At the same time, the amplicon was digested with the ApaI and SalI restriction enzymes and ligated to a similarly digested pMTnPac plasmid to create the pTnPacCOMP140P110. This plasmid was used to reintroduce the wild-type alleles of the MG_191 and MG_192 genes to the G37ΔAdh mutant. The COMmg192-F (ApaI) primer includes the upstream region (70 nucleotides) of the MG_191 gene, which contains a strong promoter identified in a previous study [47]. This promoter was used to drive the transcription of the transposon-encoded copy of the MG_191 and MG_192 genes in all mutants.

To generate P140 and P110 variants carrying specific mutations in the Engelman motifs, the target mutations in the MG_191 and or MG_192 genes were introduced by Exsite-PCR using the pBE-COMP140P110 as a template and the specific primers for each mutant (S8 Table). Then, plasmids containing the P140 and P110 alleles with the desired mutations were re-ligated and mutant P140 and P110 alleles excised by digestion with ApaI and SalI restriction enzymes. Finally, DNA fragments with mutant alleles were ligated into a pMTnPac plasmid previously digested with ApaI and SalI, to generate the corresponding pMTnPacCOMP140P110 plasmid series.

Sequencing analysis of the different TnPacCOMP140P110 constructs using primers Tnp3, RTPCR192-F, RTPCR192-R, and PacUp, ruled out the presence of additional mutations in the MG_191 and MG_192 sequences. These plasmids were transformed into the *M. genitalium* G37∆Adh null mutant to create the different P140 and P110 variant strains. Identification of the minitransposon insertion site in the individual clones was done by sequencing using the PacDown primer and chromosomal DNA as a template.

### Transformation and screening

*M. genitalium* G37∆Adh null mutants were transformed by electroporation using 10 μg of plasmid DNA of the different minitransposons or 30 μg when using plasmids for gene replacement experiments, as previously described [45,48]. Puromycin-resistant colonies were picked, propagated, and stored at –80 °C. For screening purposes, strains were further propagated in antibiotic supplemented SP4 medium in 25 cm^2^ tissue culture flasks. To obtain crude lysates from grown cultures, SP4 medium was removed and cells were lysed using 50 μL of Lysis Buffer (0.1 M Tris-HCl pH 8.5, 0.05% Tween-20, and 250 μg mL^−1^ Proteinase K) and incubated for 1 h at 37 °C. Then, Proteinase K was inactivated at 96 °C for 10 min. *M. genitalium* lysates were screened by PCR or direct Sanger sequencing using the primers in S8 Table.

### Sequencing reactions

DNA sequencing reactions were performed using BigDye v3.1 Cycle Sequencing kit using 2.5 μL of genomic DNA or *M. genitalium* lysate, following manufacturer’s instructions (Thermo Fisher Scientific). All reactions were analysed in an ABI PRISM 3130xl Genetic Analyzer at the Servei de Genòmica i Bioinformàtica (UAB).

### SDS-PAGE and western blotting

Whole-cell lysates were obtained from mid-log phase cultures grown in 75 cm^2^ flasks. Protein concentration was determined with the Pierce BCA Protein Assay Kit (Thermo Fisher Scientific), and similar amounts of total protein were separated by SDS-PAGE following standard procedures.

For specific detection of *M. genitalium* proteins, SDS-PAGE gels were electrotransferred to PVDF membranes by a Semi-dry transfer System, using cold Towbin buffer (25 mM Tris, 192 mM Glycine pH 8.3-8.4, 20% Methanol (v/v)) following the standard protocols [49,50]. Membranes were probed with the appropriate primary antibody in blocking solution, which was then detected using a secondary antibody conjugated with horseradish peroxidase (Bio-Rad). Antibodies used in this study are listed in S9 Table. Bioluminescence reaction was catalyzed with *Luminata Forte Western HRP substrate* (Merk Millipore). Visualization and image optimization was performed in a VersaDoc Imaging System (Bio-Rad) using QuantityOne software (Bio-Rad).

### Quantitative hemadsorption assay

Hemadsorption was quantified using flow cytometry as previously described [29] with few modifications. We used 10^9^ mycoplasma cells for the hemadsorption assay. Fluorescence-activated cell sorting (FACS) data were acquired using a FACSCalibur (Becton Dickinson, Franklin Lakes, USA) equipped with an air-cooled 488 nm argon laser and a 633 nm red diode laser and analysed with the CellQuest-Pro and FACSDiva software (Becton Dickinson). Binding of mycoplasma cells to red blood cells was modelled in an inverse Langmuir isothermal kinetic function:


Mf=1−Bmax[RBC]Kd+[RBC]

Plots represent the best-fitting curves to a series of hemadsorption measurements obtained from at least two biological repeats for each strain. We performed a double-gating strategy, using a preliminary FL3-H/FL2-H gate following an SSC-H/FL1-H gate.

### Time lapse micro-cinematography

Effects of antibody sera on mycoplasma cells were investigated by time lapse cinematography of *M. pneumoniae* cells growing in IBIDI 8-well chamber slides using SP4 medium supplemented with 3. Prior to observation, the medium was replaced with fresh SP4 supplemented with 3% gelatin and pre-warmed at 37°C. After incubation for 10 minutes at 37 ºC and 5% CO_2_, the slide was placed in a Nikon Eclipse TE 2000-E inverted microscope equipped with a Microscope Cage Incubation System (Okolab) at 37 ºC. Images were captured at 0.5 s intervals for a total observation time of 10 min, and the different antibodies were dispensed directly into the wells after the first 50 seconds of observation. Frequencies of motile cells and detached cells before adding the different antibodies were calculated from the images collected between 0 and 50 seconds of observation. Frequencies of motile cells and detached cells after adding the different antibodies were calculated from the pictures collected between 50 seconds and 10 minutes of observation.

### Immunofluorescence microscopy

The immunofluorescence staining of mycoplasma cells on chamber slides was similar to previously described [14,27], with several modifications. All mycoplasma cells were washed with PBS containing 0.02% Tween 20 (PBS-T) except cells from *M. genitalium* G37∆Adh strain and Engelman motif mutant strains, which were washed in PBS containing 136 mM sucrose (PBS-Suc), prewarmed at 37 °C. Then, each well was fixed with 200 μL of 3% paraformaldehyde (wt/vol) and 0.1% glutaraldehyde. Cells were washed three times with PBS-T (or PBS-Suc), and slides were immediately treated with 3% BSA in PBS-T (blocking solution) for 30 minutes. The blocking solution was removed and each well was incubated for 1 hour with 200 μL of the primary antibodies diluted in blocking solution. We have used a 1/2000 dilution for all polyclonal sera. Wells were washed three times with PBS-T or PBS-Suc and incubated for 1 hour with a 1/2000 dilution of a goat anti-mouse Alexa 555 secondary antibody (Invitrogen) in blocking solution. Wells were then washed three times with PBS-T or PBS-Suc and incubated for 20 minutes with 100 μL of a solution of Hoechst 33342 10 μg μL^−1^ in PBS-T or PBS-Suc. Wells were finally washed once with PBS-T and replenished with 200 μL of PBS-T. Cells were examined by phase contrast and epifluorescence in an Eclipse TE 2000-E inverted microscope (Nikon). Phase contrast images, 4’,6-diamidino-2-phenylindole (DAPI, excitation 387/11 nm, emission 447/60 nm) and Texas Red (excitation 560/20 nm, emission 593/40 nm) epifluorescence images were captured with an Orca Fusion camera (Hamamatsu) controlled by NIS-Elements BR software (Nikon).

### Scanning electron microscopy

For the Scanning Electron Microscopy (SEM) analysis, the *M. genitalium* cultures were grown to mid-long phase over glass and round coverslips in a 24-well plate with fresh SP4. For non-adherent mutant strains, the coverslips were treated in advance with 0.2 mg/mL Poly-L-Lysine to allow the cells to stick to the surface of the glass. The inoculum depended on the stock concentration and usually ranged between 5-10 µL of *M. genitalium* strains. The *Mge*-WT and the mutant strains were grown overnight and washed twice with 1 mL 0.1M PB. Then, the cells were fixed with 2% paraformaldehyde and 2.5% glutaraldehyde (v/v) dissolved in 0.1M PB for two hours in the dark and room temperature. Samples were then washed again with 0.1M PB and dehydrated sequentially with increasing ethanol solutions (25, 50, 75 and 100% ethanol), 10 minutes for each solution. Once in 100% ethanol, samples were sent to Servei de Microscòpia (UAB) and immediately critical-point dried in a *K850 Critical Point Dryer* (Emitech) and then they were sputter coated with gold. Samples were observed using a MERLIN FE-SEM microscope (ZEISS).

## Supporting information

S1 Fig Nucleotides and amino-acids sequences corresponding to the Heavy and the (two) Light chains from Mab P1/MCA4.
(PDF)

S2 Fig Analysis by MALS of samples containing Mab P1/MCA4 and the C-domain constructs from P1. In each of the two samples containing the Mab P1/MCA4 and a construct of the C-domain from P1 is clear the presence of a complex with molecular weight of ~179.4 and 189.5 kDa, for constructs A1400 (a) and K1376 (b), respectively.
(PDF)

S3 Fig P1 epitope mapping of Mab P1/MCA4. A) Different constructs containing C-term trimmed versions (1 to 10) or N-term trimmed versions (11 to 18) of P1 and the minimal amino acid sequence (in red) needed to detect P1 by Mab P1/MCA4. B) Western blot analyses of the different constructs using Mab P1/MCA4. The + symbol denotes that a particular construct is detected by Mab P1/MCA4.
(PDF)

S4 Fig Western blotting analysis performed using Mab P1/MCA4 and different constructs from P1 (*M. pneumoniae*) and from P140 (*M. genitalium*). 1) *M. pneumoniae* ; 2) P1Glob. Thr29-Ala1375; 3) P1 C-terminal A1400-D1521; 4) P1 C-terminal K1376-D1521; 5) C-terminal P140 S1244-D1351. The key peptide in the epitope of P1 (1426 TDLFDPVTMLVYD 1438) presents a high sequence identity with the corresponding peptide in P140 (1270 TELFDPNTMFVYD 1282).
(PDF)

S5 Fig Map quality of the P1-Fab(P1/MCA4) complex: Identification of solvent molecules. a) Two 90° apart views of the whole cryo-EM map of the P1-Fab(P1/MCA4) complex. The map is crispy, with well-defined side chains, for most of P1 and also for the Fab variable module (VL-VH). Inset shows the map at the epitope-paratope interface (same color code as in Fig 1a). **b)** The quality of the map allowed the identification of a large number of solvent molecules in the N-terminal domain of P1.
(PDF)

S6 Fig Superposition of P1 structures. Two 90° apart views of the superposition of the P1 structures determined by X-ray crystallography (PDB code 6RC9) and in this work in the complex P1-Fab(P1/MCA4) (in brown and green, respectively).
(PDF)

S7 Fig Docking calculations on the binding interface between the Fab and the C-domain of P1. a) Interface observed in the 2.3 Å resolution map. (b) Top-ranked model obtained from HADDOCK1 docking simulations performed with a lower resolution map, the P1 crystal structure and the AlphaFold2 prediction of the Fab. Docking models were ranked based on Haddock docking score (-66.44 arbitrary units), buried surface area (1457 Å^2^), and correlation with the cryo-EM map (0.85, as calculated with Chimera^3^). The C-domain of P1 is colored in green, the loop Val1425-Asp1438 in orange, and the heavy and light chains of the Fab in cyan and magenta, respectively. For clarity, only the variable fragment of the Fab is shown.
(PDF)

S8 Fig Sequence alignments of transmembrane helices from the adhesins of species belonging to the *M.* *pneumonia* *e* cluster of mycoplasmas. Orthologues from P1 (a) and from P40/P90 (b).
(PDF)

S9 Fig SDS-PAGE with protein extracts from *M. genitalium* mutant strains. 1) Mge-WT (G37); 2) G37 ∆Adh; 3) G37 ∆Adh::COM (E1;E2;E3); 4) G37 ∆Adh::COM (E1); 5) G37 ∆Adh::COM (MutE2;MutE3); 6) G37 ∆Adh::COM (E2); 7) G37 ∆Adh::COM (E3); 8) G37 ∆Adh::COM (E1;E2); 9) G37 ∆Adh::COM (E1;E3).
(PDF)

S10 Fig Immunolabeling for the localization of the Nap complexes in *M. genitalium* WT and MutE1-E2-E3 cells. Phase contrast (PhC) and epifluorescence microscopy images of cells stained with Hoechst 33342 (in blue) and immunolabelled with a polyclonal antiserum against native Nap complexes purified from M. genitalium and goat anti-mouse Alexa 555 secondary antibody (TRITC, in red). M, merging of pictures from Hoechst 33342 staining and Nap complex immunolabelling.
(PDF)

S11 Fig C-domain of P1 movements during the “open” to “closed” transition. Top and side views (upper and lower panels, respectively) of the Nap complex ectodomains in the “open” and “closed” conformations (left and right panels, respectively). The MCA4 epitope in P1 is highlighted using red balls. Values correspond to the distances (in that perspective) between the ends of the C-domains of the two P1 subunits in the Nap complex. Besides the linear displacements the C-domain experiences a hinge rotation of about 175º.
(PDF)

S1 Table MCA Monoclonal antibodies generated against the rP1 antigen (A1160-Q1518).
(PDF)

S2 Table Cryo-EM data collection of the P1-Fab complex and model refinement of the P1-Fab complex.
(PDF)

S3 Table Monoclonal/polyclonal antibodies inhibition assays (extended data).
(PDF)

S4 Table Engelman motif mutants obtained.
(PDF)

S5 Table Quantification of the frequency of cells with multiple terminal organelles (mTO) and cells with no terminal organelle for each strain.
(PDF)

S6 Table Primers used for P1 and P40/P90 protein constructs expression.
(PDF)

S7 Table Plasmids used for expression of P1 protein fragments for epitope mapping of P1/MCA4.
(PDF)

S8 Table Primers used in the generation of *M. genitalium* adhesins constructs.
(PDF)

S9 Table Antibodies used in this work.
(PDF)

S1 Video Microcinematography of *M. pneumoniae* cells in the presence of P1/MCA4 monoclonal antibody.
The clock counter timer at the top-left side of the movie shows the actual microcinematography time in mm:ss format (m: minutes; s: seconds). P1/MCA4 at a final concentration of 10 μg mL^−1^ was added to the culture medium at minute 1:10. This microcinematography was performed with no gelatin added to the culture medium.
(AVI)

S2 Video Microcinematography of *M. pneumoniae* cells in the presence of P1 ectodomain polyclonal antisera.
Mycoplasma cells were in the presence of SP4 medium supplemented with 3% gelatin. Frames were taken each 0.5 seconds of observation and are showed at 20 frames per second in the movie. The blank frame/s in the first seconds of the movie denote when the polyclonal antisera was added to the cells.
(MP4)

S3 Video Microcinematography of *M. pneumoniae* cells in in the presence P1 N-term polyclonal antisera.
Mycoplasma cells were in the presence of SP4 medium supplemented with 3% gelatin. Frames were taken each 0.5 seconds of observation and are showed at 20 frames per second in the movie. The blank frame/s in the first seconds of the movie denote when the polyclonal antisera was added to the cells.
(MP4)

S4 Video Microcinematography of *M. pneumoniae* cells in in the presence of P40/P90 ectodomain polyclonal antisera.
Mycoplasma cells were in the presence of SP4 medium supplemented with 3% gelatin. Frames were taken each 0.5 seconds of observation and are showed at 20 frames per second in the movie. The blank frame/s in the first seconds of the movie denote when the polyclonal antisera was added to the cells.
(MP4)

S5 Video Modeling the GPCA attachment and detachment cycle and halting of the cycle by the interaction with a Fab. The video first shows the side and top views of the GPCA complex transitioning from an open conformation (where sialic acid binding is possible) to a closed conformation (sialic acid release). In this transition, the displacement of the transmembrane helix is linked to the hinge rotation of the C-terminal domain from P1. Moreover, the N-terminal domain also moves interacting tightly with the N-domain of P40/P90, which forces the release of the sialic acid from its binding site. This attachment and detachment cycle is interrupted when a Fab interacts with P1 and traps the closed conformation of the C-domain from P1, impeding the complex to reach again the open conformation.
(MP4)

## References

[1] Waites KB, Xiao L, Liu Y, Balish MF, Atkinson TP. *Mycoplasma pneumoniae* from the respiratory tract and beyond. Clin Microbiol Rev. 2017;30(3):747–809. doi: 10.1128/CMR.00114-16 28539503 PMC5475226

[2] Parrott GL, Kinjo T, Fujita J. A compendium for *Mycoplasma pneumoniae*. Front Microbiol. 2016;7:513. doi: 10.3389/fmicb.2016.00513 27148202 PMC4828434

[3] Rangroo R, Young M, Davis A, Pack S, Thakore S, Schepcoff A, et al. the severity of the co-infection of *Mycoplasma pneumoniae* in COVID-19 patients. Cureus. 2022;14(4):e24563. doi: 10.7759/cureus.24563 35664402 PMC9148197

[4] Meyer Sauteur PM, Beeton ML, European Society of Clinical Microbiology and Infectious Diseases (ESCMID) Study Group for Mycoplasma and Chlamydia Infections (ESGMAC), and the ESGMAC *Mycoplasma pneumoniae* Surveillance (MAPS) study group. Pneumonia outbreaks due to re-emergence of *Mycoplasma pneumoniae*. Lancet Microbe. 2024;5(6):e514. doi: 10.1016/S2666-5247(23)00406-8 38342111

[5] Mazzolini R, Rodríguez-Arce I, Fernández-Barat L, Piñero-Lambea C, Garrido V, Rebollada-Merino A, et al. Engineered live bacteria suppress *Pseudomonas aeruginosa* infection in mouse lung and dissolve endotracheal-tube biofilms. Nat Biotechnol. 2023;41(8):1089–98. doi: 10.1038/s41587-022-01584-9 36658340 PMC10421741

[6] Taylor-Robinson D. *Mycoplasma genitalium* – an up-date. Int J STD AIDS. 2002;13(3):145–51. doi: 10.1258/0956462021924776 11860689

[7] Raj JS, Rawre J, Dhawan N, Khanna N, Dhawan B. *Mycoplasma genitalium*: a new superbug. Indian J Sex Transm Dis AIDS. 2022;43(1):1–12. doi: 10.4103/ijstd.ijstd_103_20 35846530 PMC9282694

[8] Henderson GP, Jensen GJ. Three-dimensional structure of *Mycoplasma pneumoniae*’s attachment organelle and a model for its role in gliding motility. Mol Microbiol. 2006;60(2):376–85. doi: 10.1111/j.1365-2958.2006.05113.x 16573687

[9] Miyata M, Hamaguchi T. Integrated information and prospects for gliding mechanism of the pathogenic bacterium *Mycoplasma pneumoniae*. Front Microbiol. 2016;7:960. doi: 10.3389/fmicb.2016.00960 27446003 PMC4923136

[10] Seybert A, Herrmann R, Frangakis AS. Structural analysis of *Mycoplasma pneumoniae* by cryo-electron tomography. J Struct Biol. 2006;156(2):342–54. doi: 10.1016/j.jsb.2006.04.010 16875842

[11] Leith DK, Hansen EJ, Wilson RM, Krause DC, Baseman JB. Hemadsorption and virulence are separable properties of *Mycoplasma pneumoniae*. Infect Immun. 1983;39(2):844–50. doi: 10.1128/iai.39.2.844-850.1983 6403462 PMC348026

[12] Szczepanek SM, Majumder S, Sheppard ES, Liao X, Rood D, Tulman ER, et al. Vaccination of BALB/c mice with an avirulent *Mycoplasma pneumoniae* P30 mutant results in disease exacerbation upon challenge with a virulent strain. Infect Immun. 2012;80(3):1007–14. doi: 10.1128/IAI.06078-11 22252865 PMC3294651

[13] Prince OA, Krunkosky TM, Krause DC. In vitro spatial and temporal analysis of *Mycoplasma pneumoniae* colonization of human airway epithelium. Infect Immun. 2014;82(2):579–86. doi: 10.1128/IAI.01036-13 24478073 PMC3911394

[14] Nakane D, Kenri T, Matsuo L, Miyata M. Systematic structural analyses of attachment organelle in *Mycoplasma pneumoniae*. PLoS Pathog. 2015;11(12):e1005299. doi: 10.1371/journal.ppat.1005299 26633540 PMC4669176

[15] Kawamoto A, Matsuo L, Kato T, Yamamoto H, Namba K, Miyata M. Periodicity in attachment organelle revealed by electron cryotomography suggests conformational changes in gliding mechanism of *Mycoplasma pneumoniae*. mBio. 2016;7(2):e00243-16. doi: 10.1128/mBio.00243-16 27073090 PMC4959525

[16] Mizutani M, Sasajima Y, Miyata M. Force and stepwise movements of gliding motility in human pathogenic bacterium *Mycoplasma pneumoniae*. Front Microbiol. 2021;12:747905. doi: 10.3389/fmicb.2021.747905 34630372 PMC8498583

[17] Krause DC, Leith DK, Wilson RM, Baseman JB. Identification of *Mycoplasma pneumoniae* proteins associated with hemadsorption and virulence. Infect Immun. 1982;35(3):809–17. doi: 10.1128/iai.35.3.809-817.1982 6802761 PMC351120

[18] Scheffer MP, Gonzalez-Gonzalez L, Seybert A, Ratera M, Kunz M, Valpuesta JM, et al. Structural characterization of the NAP; the major adhesion complex of the human pathogen *Mycoplasma genitalium*. Mol Microbiol. 2017;105(6):869–79. doi: 10.1111/mmi.13743 28671286

[19] Vizarraga D, Kawamoto A, Matsumoto U, Illanes R, Pérez-Luque R, Martín J, et al. Immunodominant proteins P1 and P40/P90 from human pathogen *Mycoplasma pneumoniae*. Nat Commun. 2020;11(1):5188. doi: 10.1038/s41467-020-18777-y 33057023 PMC7560827

[20] Vizarraga D, Torres-Puig S, Aparicio D, Pich OQ. The sialoglycan binding adhesins of *Mycoplasma genitalium* and *Mycoplasma pneumoniae*. Trends Microbiol. 2021;29(6):477–81. doi: 10.1016/j.tim.2021.01.011 33593698

[21] Williams CR, Chen L, Sheppard ES, Chopra P, Locklin J, Boons G-J, et al. Distinct *Mycoplasma pneumoniae* Interactions with sulfated and sialylated receptors. Infect Immun. 2020;88(11):e00392-20. doi: 10.1128/IAI.00392-20 32839185 PMC7573437

[22] Aparicio D, Scheffer MP, Marcos-Silva M, Vizarraga D, Sprankel L, Ratera M, et al. Structure and mechanism of the Nap adhesion complex from the human pathogen *Mycoplasma genitalium*. Nat Commun. 2020;11(1):2877. doi: 10.1038/s41467-020-16511-2 32513917 PMC7280502

[23] Aparicio D, Torres-Puig S, Ratera M, Querol E, Piñol J, Pich OQ, et al. *Mycoplasma genitalium* adhesin P110 binds sialic-acid human receptors. Nat Commun. 2018;9(1):4471. doi: 10.1038/s41467-018-06963-y 30367053 PMC6203739

[24] Sprankel L, Scheffer MP, Manger S, Ermel UH, Frangakis AS. Cryo-electron tomography reveals the binding and release states of the major adhesion complex from *Mycoplasma genitalium*. PLoS Pathog. 2023;19(11):e1011761. doi: 10.1371/journal.ppat.1011761 37939157 PMC10659161

[25] Russ WP, Engelman DM. The GxxxG motif: a framework for transmembrane helix-helix association. J Mol Biol. 2000;296(3):911–9. doi: 10.1006/jmbi.1999.3489 10677291

[26] Teese MG, Langosch D. Role of GxxxG motifs in transmembrane domain interactions. Biochemistry. 2015;54(33):5125–35. doi: 10.1021/acs.biochem.5b00495 26244771

[27] Sprankel L, Vizarraga D, Martín J, Manger S, Meier-Credo J, Marcos M, et al. Essential protein P116 extracts cholesterol and other indispensable lipids for mycoplasmas. Nat Struct Mol Biol. 2023;30(3):321–9. doi: 10.1038/s41594-023-00922-y 36782049 PMC10023570

[28] Seto S, Kenri T, Tomiyama T, Miyata M. Involvement of P1 adhesin in gliding motility of *Mycoplasma pneumoniae* as revealed by the inhibitory effects of antibody under optimized gliding conditions. J Bacteriol. 2005;187(5):1875–7. doi: 10.1128/JB.187.5.1875-1877.2005 15716461 PMC1064011

[29] García-Morales L, González-González L, Costa M, Querol E, Piñol J. Quantitative assessment of *Mycoplasma* hemadsorption activity by flow cytometry. PLoS One. 2014;9(1):e87500. doi: 10.1371/journal.pone.0087500 24498118 PMC3907496

[30] Pich OQ, Burgos R, Querol E, Piñol J. P110 and P140 cytadherence-related proteins are negative effectors of terminal organelle duplication in *Mycoplasma genitalium*. PLoS One. 2009;4(10):e7452. doi: 10.1371/journal.pone.0007452 19829712 PMC2759538

[31] Burgos R, Pich OQ, Ferrer-Navarro M, Baseman JB, Querol E, Piñol J. *Mycoplasma genitalium* P140 and P110 cytadhesins are reciprocally stabilized and required for cell adhesion and terminal-organelle development. J Bacteriol. 2006;188(24):8627–37. doi: 10.1128/JB.00978-06 17028283 PMC1698224

[32] Seybert A, Gonzalez-Gonzalez L, Scheffer MP, Lluch-Senar M, Mariscal AM, Querol E, et al. Cryo-electron tomography analyses of terminal organelle mutants suggest the motility mechanism of *Mycoplasma genitalium*. Mol Microbiol. 2018;108(3):319–29. doi: 10.1111/mmi.13938 29470847

[33] Miyata M. Centipede and inchworm models to explain *Mycoplasma* gliding. Trends Microbiol. 2008;16(1):6–12. doi: 10.1016/j.tim.2007.11.002 18083032

[34] Marseglia A, Forgione MC, Marcos-Silva M, Di Carluccio C, Manabe Y, Vizarraga D, et al. Molecular basis of bacterial lectin recognition of eukaryotic glycans: the case of *Mycoplasma pneumoniae* and *Mycoplasma genitalium* cytoadhesins. Int J Biol Macromol. 2024;279(Pt 2):135277. doi: 10.1016/j.ijbiomac.2024.135277 39226978

[35] Kasai T, Nakane D, Ishida H, Ando H, Kiso M, Miyata M. Role of binding in *Mycoplasma* mobile and *Mycoplasma pneumoniae* gliding analyzed through inhibition by synthesized sialylated compounds. J Bacteriol. 2013;195(3):429–35. doi: 10.1128/JB.01141-12 23123913 PMC3554017

[36] Mizutani M, Miyata M. Behaviors and energy source of *Mycoplasma gallisepticum* gliding. J Bacteriol. 2019;201(19):e00397-19. doi: 10.1128/JB.00397-19 31308069 PMC6755739

[37] Köhler G, Milstein C. Continuous cultures of fused cells secreting antibody of predefined specificity. J Immunol. 1975;174:2453–5. 15728446

[38] Zheng SQ, Palovcak E, Armache J-P, Verba KA, Cheng Y, Agard DA. MotionCor2: anisotropic correction of beam-induced motion for improved cryo-electron microscopy. Nat Methods. 2017;14(4):331–2. doi: 10.1038/nmeth.4193 28250466 PMC5494038

[39] Zhang K. Gctf: real-time CTF determination and correction. J Struct Biol. 2016;193(1):1–12. doi: 10.1016/j.jsb.2015.11.003 26592709 PMC4711343

[40] Zivanov J, Nakane T, Forsberg BO, Kimanius D, Hagen WJ, Lindahl E, et al. New tools for automated high-resolution cryo-EM structure determination in RELION-3. Elife. 2018;7:e42166. doi: 10.7554/eLife.42166 30412051 PMC6250425

[41] Punjani A, Rubinstein JL, Fleet DJ, Brubaker MA. cryoSPARC: algorithms for rapid unsupervised cryo-EM structure determination. Nat Methods. 2017;14(3):290–6. doi: 10.1038/nmeth.4169 28165473

[42] Emsley P, Cowtan K. Coot: model-building tools for molecular graphics. Acta Crystallogr D Biol Crystallogr. 2004;60(Pt 12 Pt 1):2126–32. doi: 10.1107/S0907444904019158 15572765

[43] Afonine PV, Poon BK, Read RJ, Sobolev OV, Terwilliger TC, Urzhumtsev A, et al. Real-space refinement in PHENIX for cryo-EM and crystallography. Acta Crystallogr D Struct Biol. 2018;74(Pt 6):531–44. doi: 10.1107/S2059798318006551 29872004 PMC6096492

[44] Torres-Puig S, Broto A, Querol E, Piñol J, Pich OQ. A novel sigma factor reveals a unique regulon controlling cell-specific recombination in *Mycoplasma genitalium*. Nucleic Acids Res. 2015;43(10):4923–36. doi: 10.1093/nar/gkv422 25925568 PMC4446450

[45] Pich OQ, Burgos R, Planell R, Querol E, Piñol J. Comparative analysis of antibiotic resistance gene markers in *Mycoplasma genitalium*: application to studies of the minimal gene complement. Microbiology (Reading). 2006;152(Pt 2):519–27. doi: 10.1099/mic.0.28287-0 16436439

[46] Mariscal AM, González-González L, Querol E, Piñol J. All-in-one construct for genome engineering using Cre-lox technology. DNA Res. 2016;23(3):263–70. doi: 10.1093/dnares/dsw015 27084897 PMC4909314

[47] Musatovova O, Dhandayuthapani S, Baseman JB. Transcriptional starts for cytadherence-related operons of *Mycoplasma genitalium*. FEMS Microbiol Lett. 2003;229(1):73–81. doi: 10.1016/S0378-1097(03)00789-4 14659545

[48] Torres-Puig S, Martínez-Torró C, Granero-Moya I, Querol E, Piñol J, Pich OQ. Activation of σ20-dependent recombination and horizontal gene transfer in *Mycoplasma genitalium*. DNA Res. 2018;25(4):383–93. doi: 10.1093/dnares/dsy011 29659762 PMC6105099

[49] Mahmood T, Yang P-C. Western blot: technique, theory, and trouble shooting. N Am J Med Sci. 2012;4(9):429–34. doi: 10.4103/1947-2714.100998 23050259 PMC3456489

[50] Towbin H, Staehelin T, Gordon J. Electrophoretic transfer of proteins from polyacrylamide gels to nitrocellulose sheets: procedure and some applications. Proc Natl Acad Sci U S A. 1979;76(9):4350–4. doi: 10.1073/pnas.76.9.4350 388439 PMC411572

