# Supplementary material for: Dynamics of the adhesion complex of the human pathogens Mycoplasma pneumoniae and Mycoplasma genitalium
Source: PLoS Pathog. 2025 Mar 28;21(3):e1012973. doi: 10.1371/journal.ppat.1012973 (PMC11984735; doi:10.1371/journal.ppat.1012973)
Supplement: S1 Fig — (PDF) [file ppat.1012973.s001.pdf]

P1/MCA4 H chain

DDBJ/ENA/GenBank accession no. LC600310

Signal peptide -FR1-CDR1-FR2-CDR2-FR3-CDR3-FR4-constant region

```
atgggatggagctggatctttctcttcctcctgtcaggaactgcaggtgtccactctgaggtccagctgcaacagtctggacctgaactggtgaagcctggaacttcaatgaagatatcc   120
M G W S W I F L F L L S G T A G V H S E V Q L Q Q S G P E L V K P G T S M K I S   40
tgcaaggcttctggttactcattcactggctacaccatgaactgggtgaagcagagccatggaaagagccttgagtggattggacttattaatccttacaatggtggtactaactacaac   240
C K A S G Y S F T G Y T M N W V K Q S H G K S L E W I G L I N P Y N G G T N Y N   80
cagaagttcaggggcacggccacattaactgtagacaagtcatccagcacagcctacatggagctcctcagtctgacatctgaggactctgcagtctattactgtgcaaggtcgaactat   360
Q K F R G T A T L T V D K S S S T A Y M E L L S L T S E D S A V Y Y C A R S N Y   120
gcttacgacctattgatggactactggggtcaaggaacctcagtcaccgtctcctcagccaaaacgacacccccatctgtctatccactggcccctggatctgctgcccaaactaactcc   480
A Y D L L M D Y W G Q G T S V T V S S A K T T P P S V Y P L A P G S A A Q T N S   160
atggtgaccctgggatgcctggtcaagggctatttccctgagccagtgacagtgacctggaactctggatccctgtccagcggtgtgcacaccttcccagctgtcctgcagtctgacctc   600
M V T L G C L V K G Y F P E P V T V T W N S G S L S S G V H T F P A V L Q S D L   200
tacactctgagcagctcagtgactgtcccctccagcacctggcccagcgagaccgtcacctgcaacgttgcccacccggccagcagcaccaaggtggacaagaaaattgtgcccagggat   720
Y T L S S S V T V P S S T W P S E T V T C N V A H P A S S T K V D K K I V P R D   240
tgtggttgtaagccttgcatatgtacagtcccagaagtatcatctgtcttcatcttccccccaaagcccaaggatgtgctcaccattactctgactcctaaggtcacgtgtgttgtggta   840
C G C K P C I C T V P E V S S V F I F P P K P K D V L T I T L T P K V T C V V V   280
gacatcagcaaggatgatcccgaggtccagttcagctggtttgtagatgatgtggaggtgcacacagctcagacgcaaccccgggaggagcagttcaacagcactttccgctcagtcagt   960
D I S K D D P E V Q F S W F V D D V E V H T A Q T Q P R E E Q F N S T F R S V S   320
gaacttcccatcatgcaccaggactggctcaatggcaaggagttcaaatgcagggtcaacagtgcagctttccctgcccccatcgagaaaaccatctccaaaaccaaaggcagaccgaag   1080
E L P I M H Q D W L N G K E F K C R V N S A A F P A P I E K T I S K T K G R P K   360
gctccacaggtgtacaccattccacctcccaaggagcagatggccaaggataaagtcagtctgacctgcatgataacagacttcttccctgaagacattactgtggagtggcagtggaat   1200
A P Q V Y T I P P P K E Q M A K D K V S L T C M I T D F F P E D I T V E W Q W N   400
gggcagccagcggagaactacaagaacactcagcccatcatggacacagatggctcttacttcgtctacagcaagctcaatgtgcagaagagcaactgggaggcaggaaatactttcacc   1320
G Q P A E N Y K N T Q P I M D T D G S Y F V Y S K L N V Q K S N W E A G N T F T   440
tgctctgtgttacatgagggcctgcacaaccaccatactgagaagagcctctcccactctcctggtaaatga   1392
C S V L H E G L H N H H T E K S L S H S P G K *   463
```

P1/MCA4 L chain 1   (L1)

DDBJ/ENA/GenBank accession no. LC600311

Signal peptide -FR1-CDR1-FR2-CDR2-FR3-CDR3-FR4-constant region

```
atgaagttgcctgttaggctgttggtgctgatgttctggattcctgcttccagcagtgatgttttgatgacccaaactccactctccctgcctgtcagtcttggagatcaagcctccatc   120
M K L P V R L L V L M F W I P A S S S D V L M T Q T P L S L P V S L G D Q A S I   40
tcttgcagatttagtcagaccattgtacatagtaatggagccacctatttagaatggtacctgcagagaccaggccagtctccaaagctcctgatctacaaagtttccaaccgattttct   240
S C R F S Q T I V H S N G A T Y L E W Y L Q R P G Q S P K L L I Y K V S N R F S   80
ggggtcccagacaggttcagtggcagtggatcagggacagatttcacactcaaaatcagcagagtggaggctgaggatctgggagtttattactgctttcaaggttcacatgttccgtgg   360
G V P D R F S G S G S G T D F T L K I S R V E A E D L G V Y Y C F Q G S H V P W   120
acgttcggtggaggcaccaagctggaaatcaaacgggctgatgctgcaccaactgtatccatcttcccaccatccagtgagcagttaacatctggaggtgcctcagtcgtgtgcttcttg   480
T F G G G T K L E I K R A D A A P T V S I F P P S S E Q L T S G G A S V V C F L   160
aacaacttctaccccaaagacatcaatgtcaagtggaagattgatggcagtgaacgacaaaatggcgtcctgaacagttggactgatcaggacagcaaagacagcacctacagcatgagc   600
N N F Y P K D I N V K W K I D G S E R Q N G V L N S W T D Q D S K D S T Y S M S   200
agcaccctcacgttgaccaaggacgagtatgaacgacataacagctatacctgtgaggccactcacaagacatcaacttcacccattgtcaagagcttcaacaggaatgagtgttag   717
S T L T L T K D E Y E R H N S Y T C E A T H K T S T S P I V K S F N R N E C *   238
```

P1/MCA4 L chain 2   (L2)

DDBJ/ENA/GenBank accession no. LC600312

Signal peptide -FR1-CDR1-FR2-CDR2-FR3-CDR3-FR4-constant region

```
atgagtcctgcccagttcctgtttctcttagtgctctggattcgggaaaccaacggtgatgttgtgatgacccagactccactcactttgtcggttaccattggacaaccagcctccatc   120
M S P A Q F L F L L V L W I R E T N G D V V M T Q T P L T L S V T I G Q P A S I   40
tcttgcaagtcaagtcagagcctcttagatagtgatggaaagacatatttgaattgcttcttacagaggccaggccagtctccaaatcgcctgatctatctggtgtctagactggactct   240
S C K S S Q S L L D S D G K T Y L N C F L Q R P G Q S P N R L I Y L V S R L D S   80
ggagtccctgacaggttcactggcagtggatcagggacagatttcacactgaaaatcagcagagtggaggctgaggatttgggagtttattattgctgtcaagttacacagtggacgttc   360
G V P D R F T G S G S G T D F T L K I S R V E A E D L G V Y Y C C Q V T Q W T F   120
ggtggaggcaccaagctggaaatcaaacgggctgatgctgcaccaactgtatccatcttcccaccatccagtgagcagttaacatctggaggtgcctcagtcgtgtgcttcttgaacaac   480
G G G T K L E I K R A D A A P T V S I F P P S S E Q L T S G G A S V V C F L N N   160
ttctaccccaaagacatcaatgtcaagtggaagattgatggcagtgaacgacaaaatggcgtcctgaacagttggactgatcaggacagcaaagacagcacctacagcatgagcagcacc   600
F Y P K D I N V K W K I D G S E R Q N G V L N S W T D Q D S K D S T Y S M S S T   200
ctcacgttgaccaaggacgagtatgaacgacataacagctatacctgtgaggccactcacaagacatcaacttcacccattgtcaagagcttcaacaggaatgagtgttag   711
L T L T K D E Y E R H N S Y T C E A T H K T S T S P I V K S F N R N E C *   236
```

Alignment of L1 and L2 amino acid sequences

```
L1  MKLPVRLLVLMFWIPASSSDVLMTQTPLSLPVSLGDQASISCRFSQTIVHSNGATYLEWYLQRPGQSPKLLIYKVSNRFSGVPDRFSGSGSGTDFTLKISRVEAEDLGVYYCFQGSHVPW  12 0
    |     |  |  ||     || |||||| | |  |  |||||  ||    | | |||   ||||||||  ||| ||   ||||||| ||||||||||||||||||||||||| |     |
L2  MSPAQFLFLLVLWIRETNGDVVMTQTPLTLSVTIGQPASISCKSSQSLLDSDGKTYLNCFLQRPGQSPNRLIYLVSRLDSGVPDRFTGSGSGTDFTLKISRVEAEDLGVYYCCQVTQ--W  118

    TFGGGTKLEIKRADAAPTVSIFPPSSEQLTSGGASVVCFLNNFYPKDINVKWKIDGSERQNGVLNSWTDQDSKDSTYSMSSTLTLTKDEYERHNSYTCEATHKTSTSPIVKSFNRNEC  238
    ||||||||||||||||||||||||||||||||||||||||||||||||||||||||||||||||||||||||||||||||||||||||||||||||||||||||||||||||||||||
    TFGGGTKLEIKRADAAPTVSIFPPSSEQLTSGGASVVCFLNNFYPKDINVKWKIDGSERQNGVLNSWTDQDSKDSTYSMSSTLTLTKDEYERHNSYTCEATHKTSTSPIVKSFNRNEC  236
```

Supplementary Figure 1. Nucleotides and amino-acids sequences corresponding to the Heavy and the (two) Light chains from Mab P1/MCA4
