# Supplementary material for: Dynamics of the adhesion complex of the human pathogens Mycoplasma pneumoniae and Mycoplasma genitalium
Source: PLoS Pathog. 2025 Mar 28;21(3):e1012973. doi: 10.1371/journal.ppat.1012973 (PMC11984735; doi:10.1371/journal.ppat.1012973)
Supplement: S2 Fig — In each of the two samples containing the Mab P1/MCA4 and a construct of the C-domain from P1 is clear the presence of a complex with molecular weight of ~179.4 and 189.5 kDa, for constructs A1400 (a) and K1376 (b), respectively. (PDF) [file ppat.1012973.s002.pdf]

a

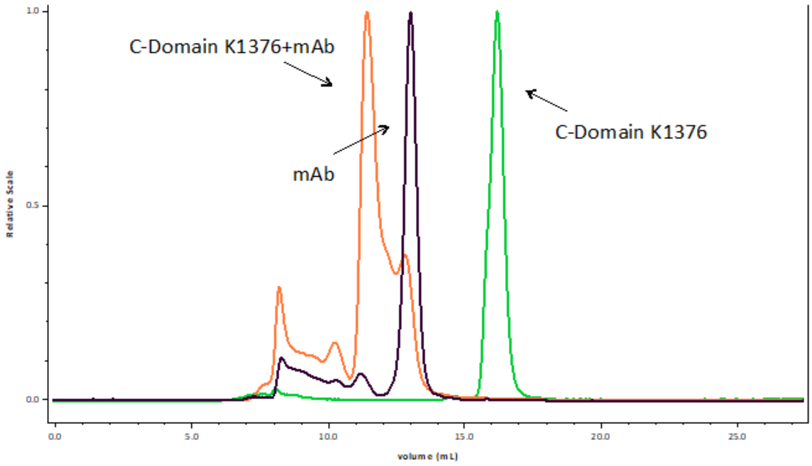

Peak	Mw (KDa)	Mass Fraction (%)
C-Domain K1376	18.92 ± 0.07	100.00
mAb	148.44 ± 0.20	100.00
C-Domain K1376+mAb	189.53 ± 0.21	67.10

b

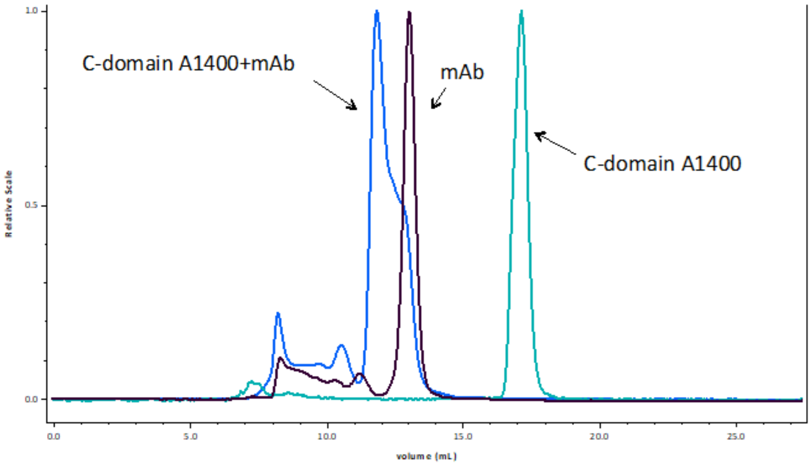

Peak	Mw (KDa)	Mass Fraction (%)
C-Domain A1376	15.79 ± 0.09	100.00
mAb	148.44 ± 0.20	100.00
C-Domain A1376+ mAb	179.44 ± 0.21	57.07

Supplementary Figure 2. Analysis by MALS of samples containing Mab P1/MCA4 and the C-domain constructs from P1. In each of the two samples containing the Mab P1/MCA4 and a construct of the C-domain from P1 is clear the presence of a complex with molecular weight of ~179.4 and 189.5 kDa, for constructs A1400 (a) and K1376 (b), respectively**.**
