# Supplementary material for: Dynamics of the adhesion complex of the human pathogens Mycoplasma pneumoniae and Mycoplasma genitalium
Source: PLoS Pathog. 2025 Mar 28;21(3):e1012973. doi: 10.1371/journal.ppat.1012973 (PMC11984735; doi:10.1371/journal.ppat.1012973)
Supplement: S3 Fig — A) Different constructs containing C-term trimmed versions (1 to 10) or N-term trimmed versions (11 to 18) of P1 and the minimal amino acid sequence (in red) needed to detect P1 by Mab P1/MCA4. B) Western blot analyses of the different constructs using Mab P1/MCA4. The + symbol denotes that a particular construct is detected by Mab P1/MCA4. (PDF) [file ppat.1012973.s003.pdf]

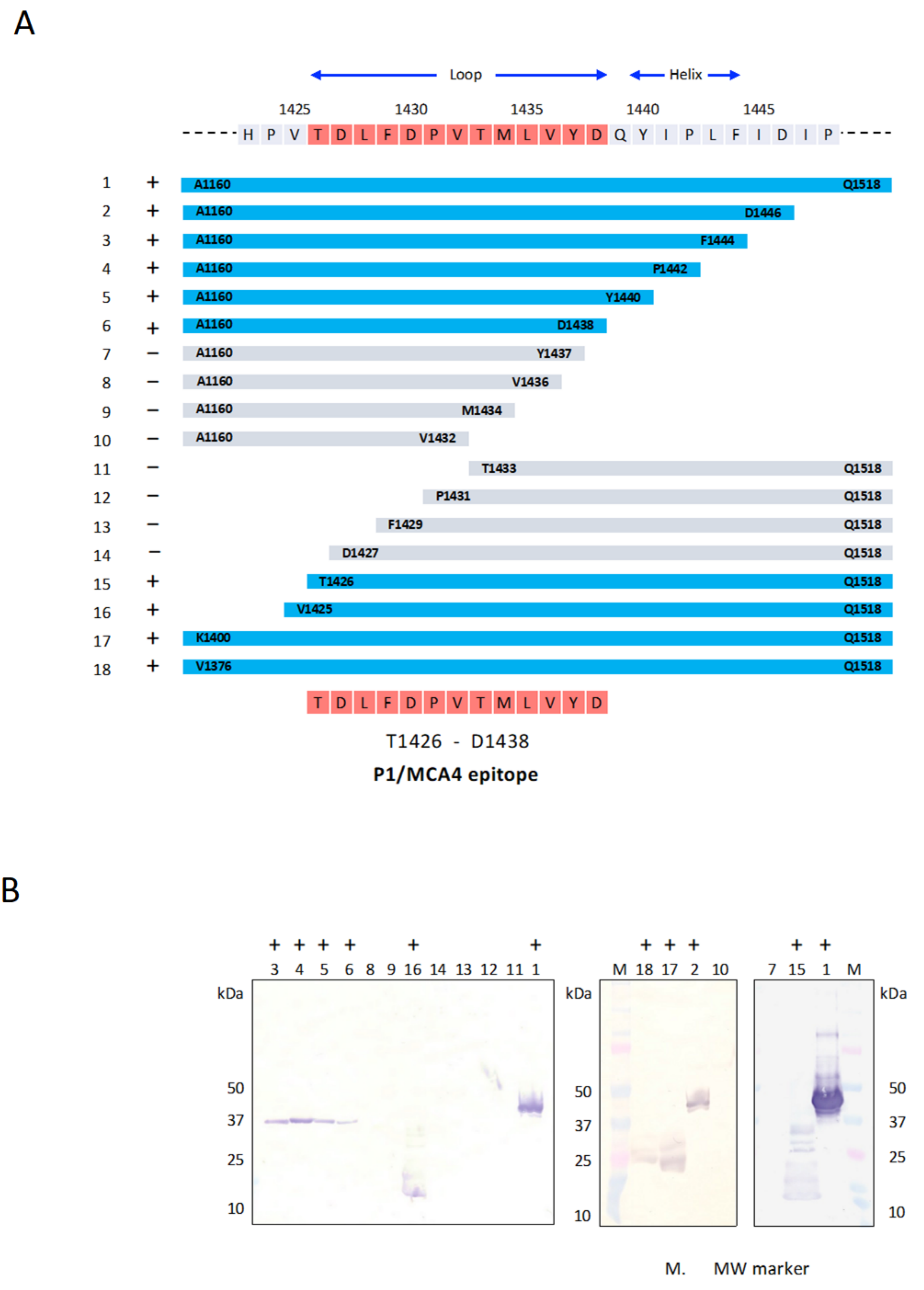

Supplementary Figure 3. P1 epitope mapping of Mab P1/MCA4. A) Different constructs containing C-term trimmed versions (1 to 10) or N-term trimmed versions (11 to 18) of P1 and the minimal amino acid sequence (in red) needed to detect P1 by Mab P1/MCA4. B) Western blot analyses of the different constructs using Mab P1/MCA4. The + symbol denotes that a particular construct is detected by Mab P1/MCA4.
