# Supplementary material for: Dynamics of the adhesion complex of the human pathogens Mycoplasma pneumoniae and Mycoplasma genitalium
Source: PLoS Pathog. 2025 Mar 28;21(3):e1012973. doi: 10.1371/journal.ppat.1012973 (PMC11984735; doi:10.1371/journal.ppat.1012973)
Supplement: S4 Fig — 1) M. pneumoniae ; 2) P1Glob. Thr29-Ala1375; 3) P1 C-terminal A1400-D1521; 4) P1 C-terminal K1376-D1521; 5) C-terminal P140 S1244-D1351. The key peptide in the epitope of P1 (1426 TDLFDPVTMLVYD 1438) presents a high sequence identity with the corresponding peptide in P140 (1270 TELFDPNTMFVYD 1282). (PDF) [file ppat.1012973.s004.pdf]

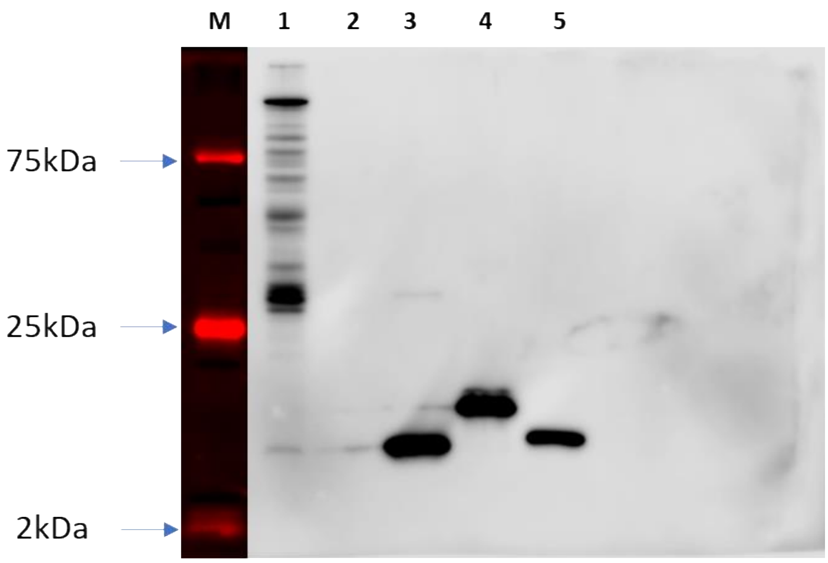

Supplementary Figure 4. Western blotting analysis performed using Mab P1/MCA4 and different constructs from P1 (*M. pneumoniae*) and from P140 (*M. genitalium*). 1) *Mpn*-WT (MPN129); 2) P1Glob. Thr29-Ala1375; 3) P1 C-terminal A1400-D1521; 4) P1 C-terminal K1376-D1521; 5) C-terminal P140 S1244-D1351. The key peptide in the epitope of P1 (1426 TDLFDPVTMLVYD 1438) presents a high sequence identity with the corresponding peptide in P140 (1270 TELFDPNTMFVYD 1282).
