# Supplementary material for: Dynamics of the adhesion complex of the human pathogens Mycoplasma pneumoniae and Mycoplasma genitalium
Source: PLoS Pathog. 2025 Mar 28;21(3):e1012973. doi: 10.1371/journal.ppat.1012973 (PMC11984735; doi:10.1371/journal.ppat.1012973)
Supplement: S5 Fig — a) Two 90° apart views of the whole cryo-EM map of the P1-Fab(P1/MCA4) complex. The map is crispy, with well-defined side chains, for most of P1 and also for the Fab variable module (VL-VH). Inset shows the map at the epitope-paratope interface (same color code as in Fig 1a). b) The quality of the map allowed the identification of a large number of solvent molecules in the N-terminal domain of P1. (PDF) [file ppat.1012973.s005.pdf]

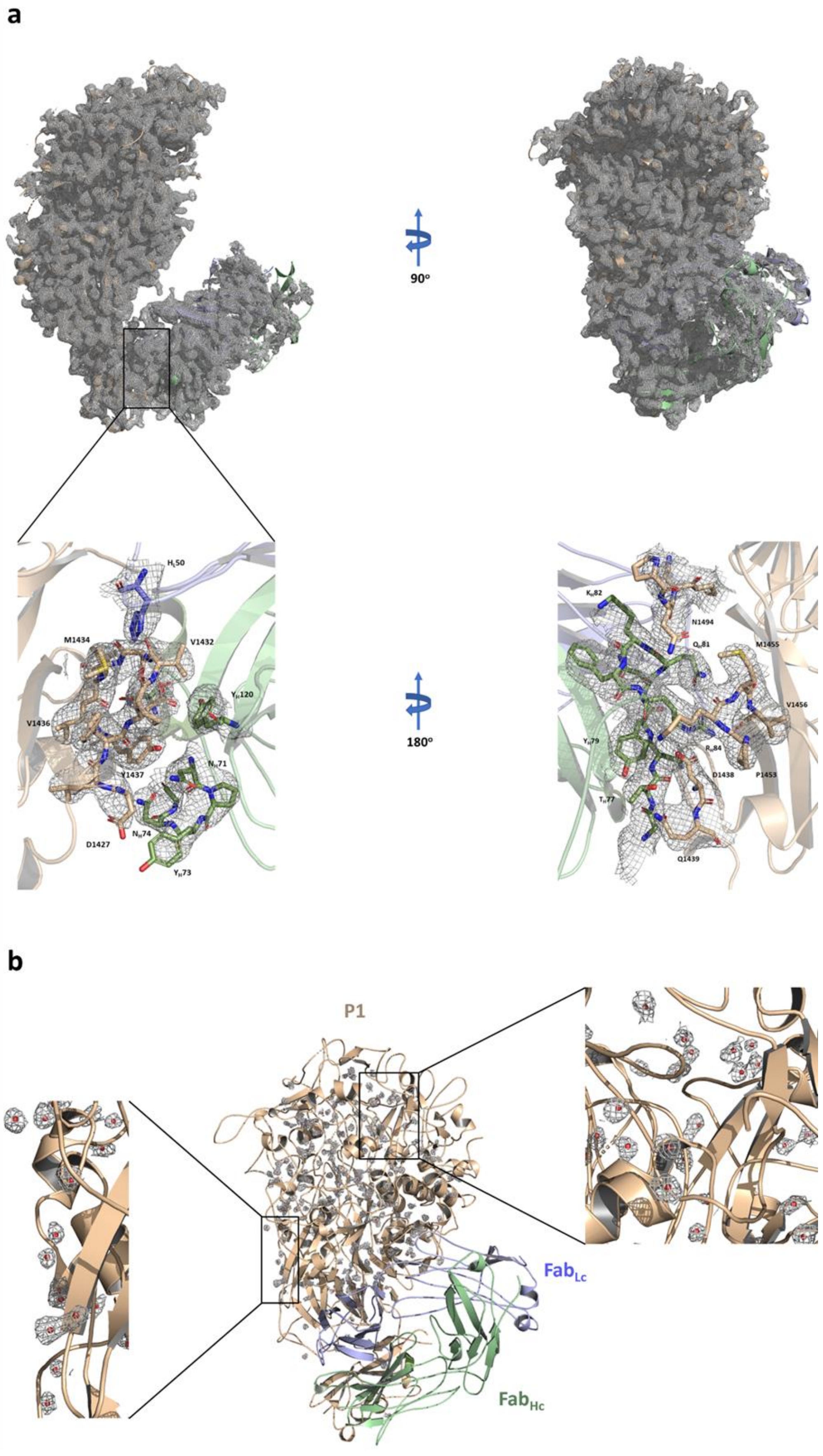
a
90°
180°
H_L50
M1434
V1432
Y_H120
V1436
Y1437
N_H71
D1427
N_H74
Y_H73
K_H82
N1494
Q_H81
M1455
V1456
Y_H79
R_H84
D1438
P1453
T_H77
Q1439
b
P1
Fab_{Lc}
Fab_{Hc}

Supplementary Figure 5. Map quality of the P1-Fab(P1/MCA4) complex: Identification of solvent molecules. a) Two 90° apart views of the whole cryo-EM map of the P1-Fab(P1/MCA4) complex. The map is crispy, with well-defined side chains, for most of P1 and also for the Fab variable module (V_L-V_H). Inset shows the map at the epitope-paratope interface (same color code as in Figure 1a). **b)** The quality of the map allowed the identification of a large number of solvent molecules in the N-terminal domain of P1.
