# Supplementary material for: Dynamics of the adhesion complex of the human pathogens Mycoplasma pneumoniae and Mycoplasma genitalium
Source: PLoS Pathog. 2025 Mar 28;21(3):e1012973. doi: 10.1371/journal.ppat.1012973 (PMC11984735; doi:10.1371/journal.ppat.1012973)
Supplement: S6 Fig — Two 90° apart views of the superposition of the P1 structures determined by X-ray crystallography (PDB code 6RC9) and in this work in the complex P1-Fab(P1/MCA4) (in brown and green, respectively). (PDF) [file ppat.1012973.s006.pdf]

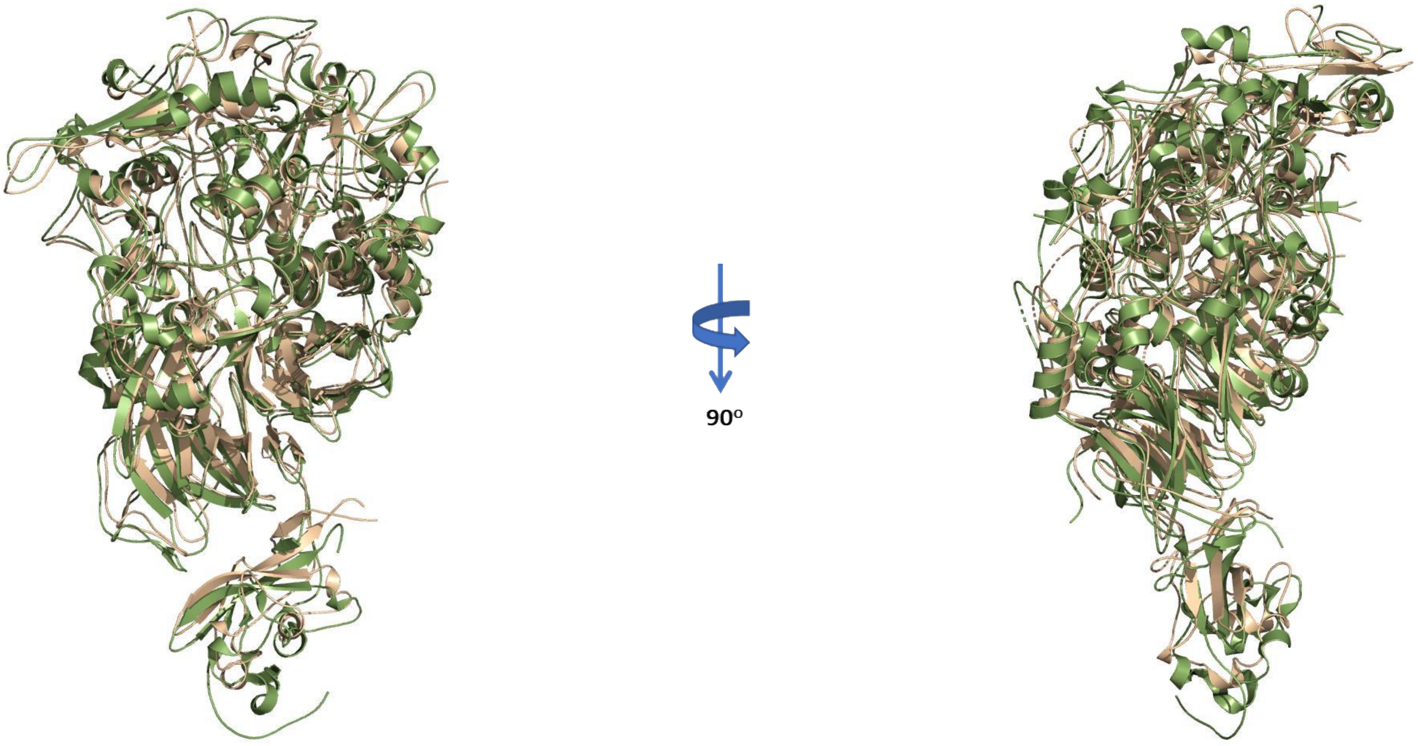

Supplementary Figure 6. Superposition of P1 structures. Two 90° apart views of the superposition of the P1 structures determined by X-ray crystallography (PDB code 6RC9) and in this work in the complex P1-Fab(P1/MCA4) (in brown and green, respectively).
