# Supplementary material for: Dynamics of the adhesion complex of the human pathogens Mycoplasma pneumoniae and Mycoplasma genitalium
Source: PLoS Pathog. 2025 Mar 28;21(3):e1012973. doi: 10.1371/journal.ppat.1012973 (PMC11984735; doi:10.1371/journal.ppat.1012973)
Supplement: S7 Fig — a) Interface observed in the 2.3 Å resolution map. (b) Top-ranked model obtained from HADDOCK1 docking simulations performed with a lower resolution map, the P1 crystal structure and the AlphaFold2 prediction of the Fab. Docking models were ranked based on Haddock docking score (-66.44 arbitrary units), buried surface area (1457 Å2), and correlation with the cryo-EM map (0.85, as calculated with Chimera3). The C-domain of P1 is colored in green, the loop Val1425-Asp1438 in orange, and the heavy and light chains of the Fab in cyan and magenta, respectively. For clarity, only the variable fragment of the Fab is shown. (PDF) [file ppat.1012973.s007.pdf]

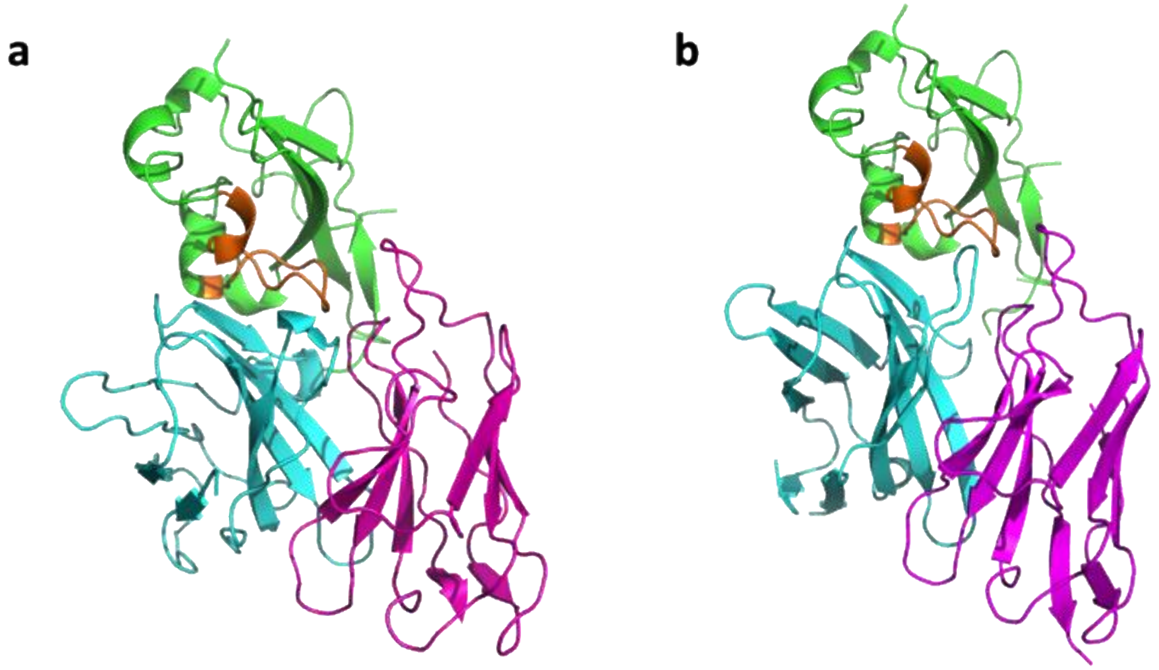

Supplementary Figure 7. Docking calculations on the binding interface between the Fab and the C-domain of P1. a) Interface observed in the 2.3 Å resolution map. (b) Top-ranked model obtained from HADDOCK[1] docking simulations performed with a lower resolution map, the P1 crystal structure and the AlphaFold2[2] prediction of the Fab. Docking models were ranked based on Haddock docking score (-66.44 arbitrary units), buried surface area (1457 Å^2), and correlation with the cryo-EM map (0.85, as calculated with Chimera[3]). The C-domain of P1 is colored in green, the loop Val1425-Asp1438 in orange, and the heavy and light chains of the Fab in cyan and magenta, respectively. For clarity, only the variable fragment of the Fab is shown.
