# Supplementary material for: Dynamics of the adhesion complex of the human pathogens Mycoplasma pneumoniae and Mycoplasma genitalium
Source: PLoS Pathog. 2025 Mar 28;21(3):e1012973. doi: 10.1371/journal.ppat.1012973 (PMC11984735; doi:10.1371/journal.ppat.1012973)
Supplement: S8 Fig — Orthologues from P1 (a) and from P40/P90 (b). (PDF) [file ppat.1012973.s008.pdf]

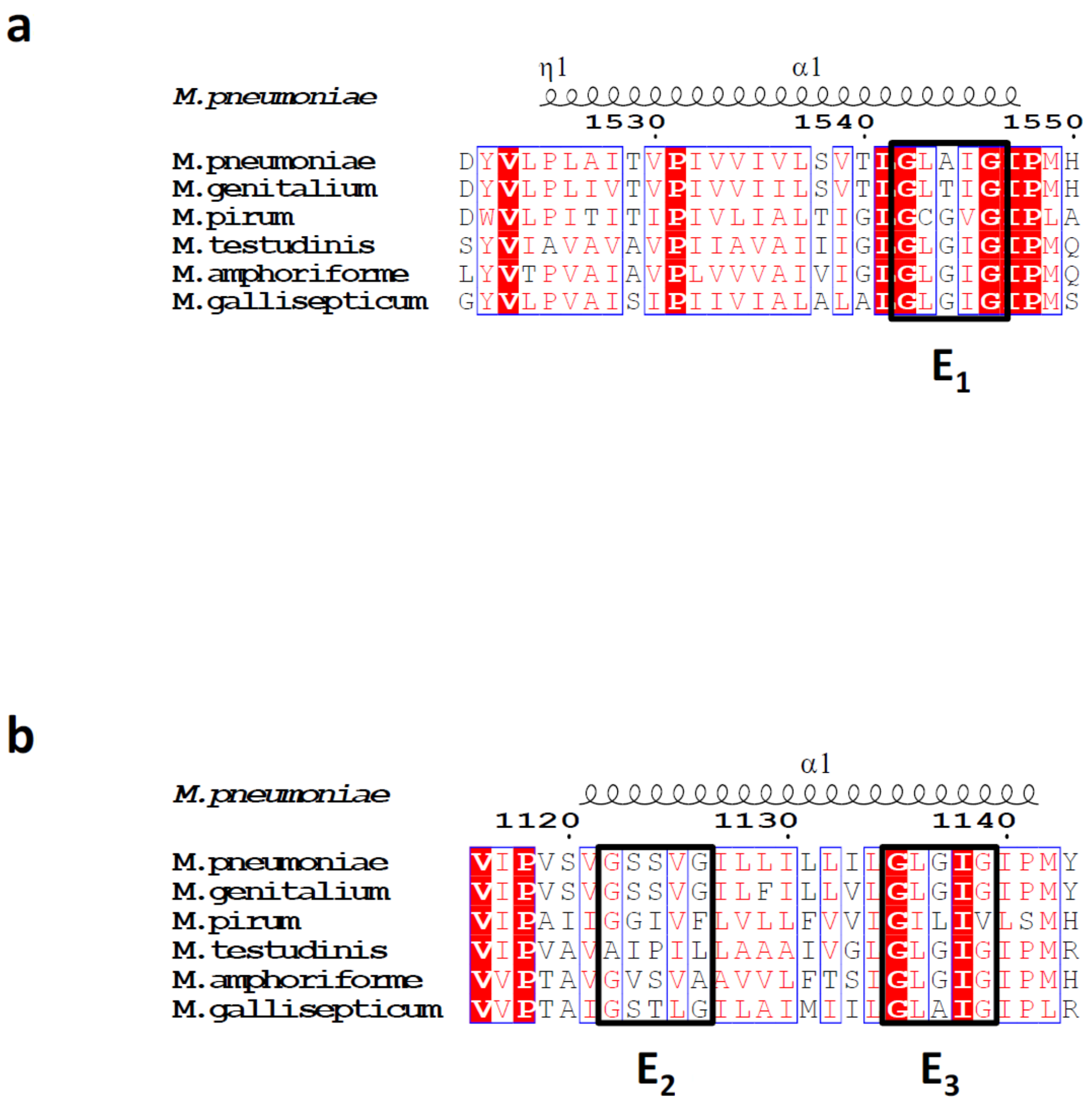

Supplementary Figure 8. Sequence alignments of transmembrane helices from the adhesins of species belonging to the pneumonia cluster of mycoplasmas. Orthologues from P1 **(a)** and from P40/P90 **(b).**
