# Supplementary material for: Dynamics of the adhesion complex of the human pathogens Mycoplasma pneumoniae and Mycoplasma genitalium
Source: PLoS Pathog. 2025 Mar 28;21(3):e1012973. doi: 10.1371/journal.ppat.1012973 (PMC11984735; doi:10.1371/journal.ppat.1012973)
Supplement: S9 Fig — 1) Mge-WT (G37); 2) G37 ∆Adh; 3) G37 ∆Adh::COM (E1;E2;E3); 4) G37 ∆Adh::COM (E1); 5) G37 ∆Adh::COM (MutE2;MutE3); 6) G37 ∆Adh::COM (E2); 7) G37 ∆Adh::COM (E3); 8) G37 ∆Adh::COM (E1;E2); 9) G37 ∆Adh::COM (E1;E3). (PDF) [file ppat.1012973.s009.pdf]

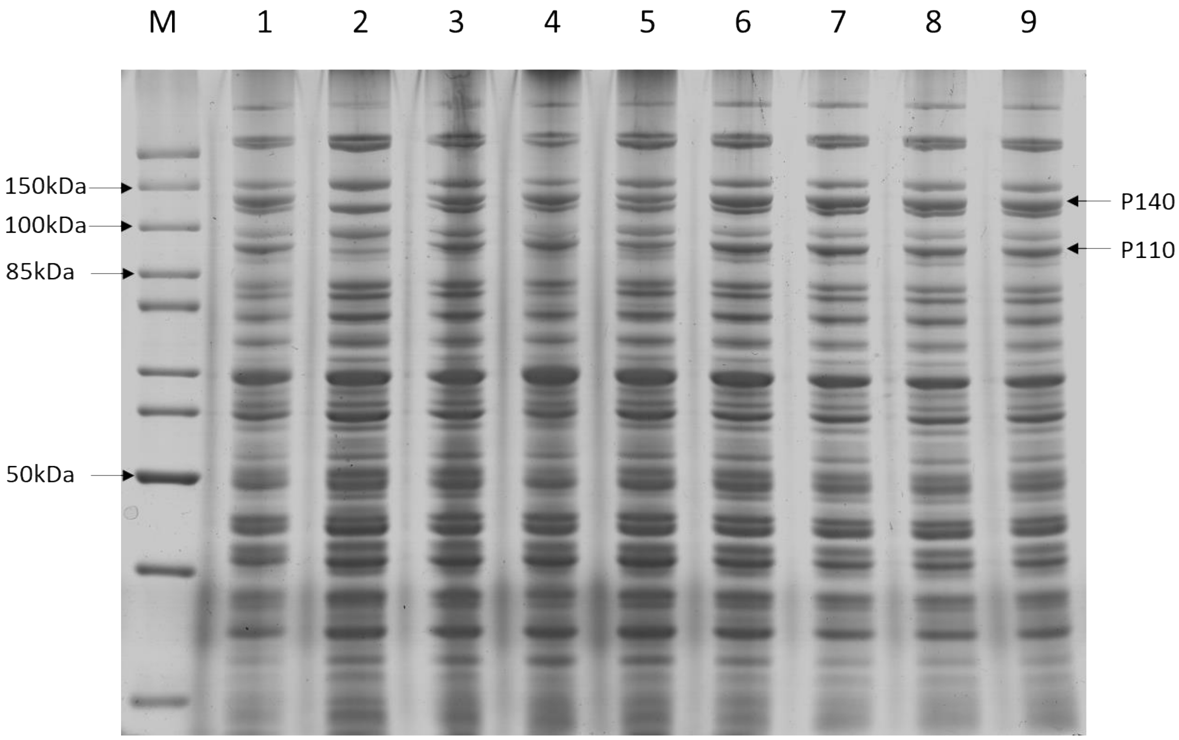

E1: G1372F-G1376F

1371Leu-**Gly**-Leu-Thr-Ile-**Gly**-Ile → 1371Leu-**Phe**-Leu-Thr-Ile-**Phe**-Ile

225680-TTG**GGA**TTAACGATT**GGA**ATT → 225680-TTG**TTT**TTAACGATT**TTT**ATT

E2: G947F-G951F

946Val-**Gly**-Ser-Ser-Val-**Gly**-Ile → 946Val-**Phe**-Ser-Ser-Val-**Phe**-Ile

228741-GTA**GGT**TCTTCAGTT**GGG**ATC → 228741-GTA**TTT**TCTTCAGTT**TTT**ATC

E3: G960F-G964F

959-Leu-**Gly**-Leu-Gly-Ile-**Gly**-Ile → 959-Leu-**Phe**-Leu-Gly-Ile-**Phe**-Ile

228780-TTA**GGA**CTTGGGATT**GGG**ATC → 228780-TTA**TTT**CTTGGGATT**TTT**ATC

Supplementary Figure 9. SDS-PAGE with protein extracts from *M. genitalium* mutant strains. 1) *Mge*-WT (G37); 2) G37 ΔAdh; 3) G37 ΔAdh::COM (E1;E2;E3); 4) G37 ΔAdh::COM (E1); 5) G37 ΔAdh::COM (MutE2;MutE3); 6) G37 ΔAdh::COM (E2); 7) G37 ΔAdh::COM (E3); 8) G37 ΔAdh::COM (E1;E2); 9) G37 ΔAdh::COM (E1;E3)
