# Supplementary material for: Dynamics of the adhesion complex of the human pathogens Mycoplasma pneumoniae and Mycoplasma genitalium
Source: PLoS Pathog. 2025 Mar 28;21(3):e1012973. doi: 10.1371/journal.ppat.1012973 (PMC11984735; doi:10.1371/journal.ppat.1012973)
Supplement: S10 Fig — Phase contrast (PhC) and epifluorescence microscopy images of cells stained with Hoechst 33342 (in blue) and immunolabelled with a polyclonal antiserum against native Nap complexes purified from M. genitalium and goat anti-mouse Alexa 555 secondary antibody (TRITC, in red). M, merging of pictures from Hoechst 33342 staining and Nap complex immunolabelling. (PDF) [file ppat.1012973.s010.pdf]

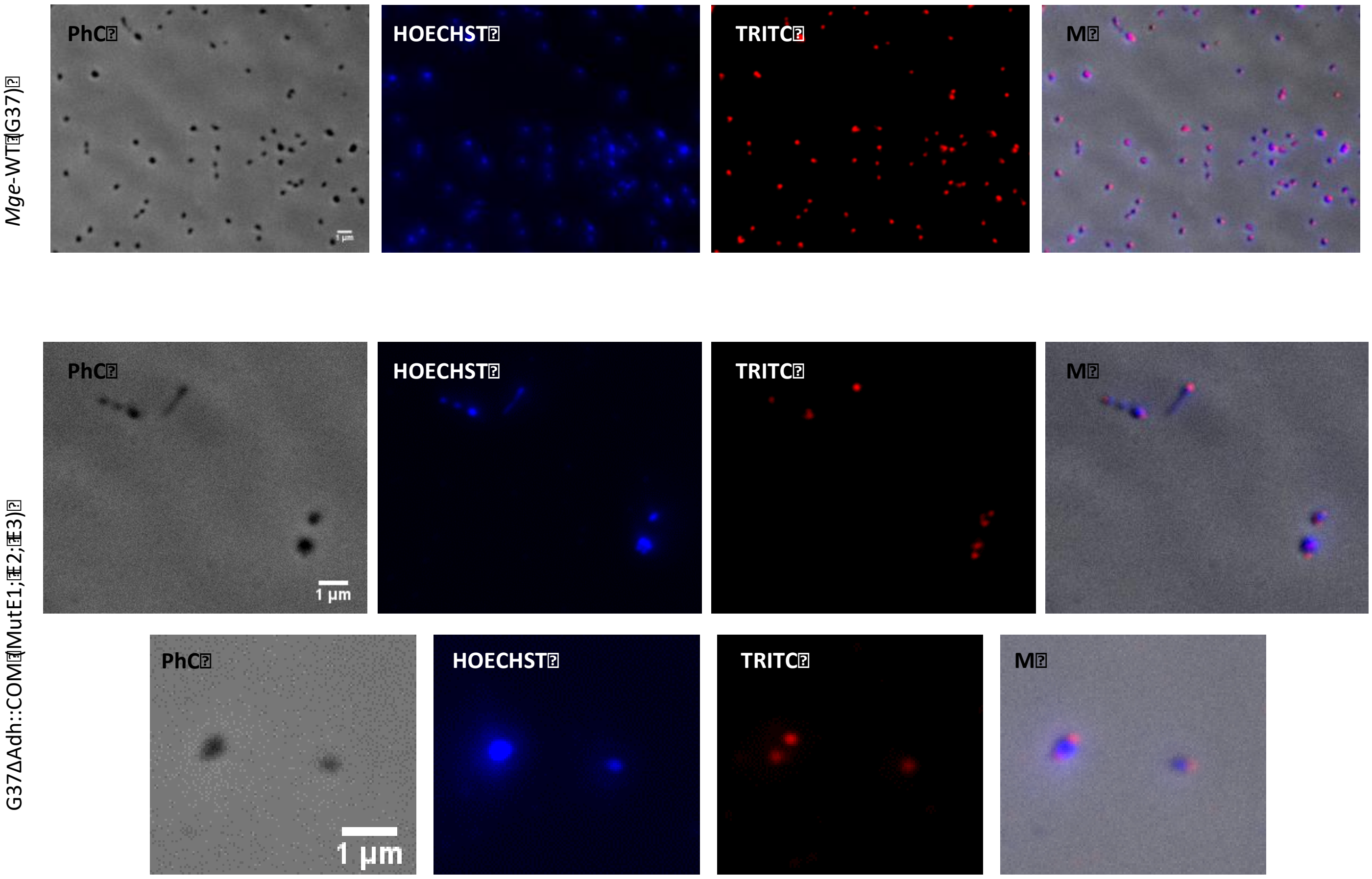

Supplementary Figure 10. Immunolabeling for the localization of the Nap complexes in *M. genitalium* WT and MutE1-E2-E3 cells. Phase contrast (PhC) and epifluorescence microscopy images of cells stained with Hoechst 33342 (in blue) and immunolabelled with a polyclonal antiserum against native Nap complexes purified from *M. genitalium and* goat anti-mouse Alexa 555 secondary antibody (TRITC, in red). M, merging of pictures from Hoechst 33342 staining and Nap complex immunolabelling.
