# Supplementary material for: Dynamics of the adhesion complex of the human pathogens Mycoplasma pneumoniae and Mycoplasma genitalium
Source: PLoS Pathog. 2025 Mar 28;21(3):e1012973. doi: 10.1371/journal.ppat.1012973 (PMC11984735; doi:10.1371/journal.ppat.1012973)
Supplement: S11 Fig — Top and side views (upper and lower panels, respectively) of the Nap complex ectodomains in the “open” and “closed” conformations (left and right panels, respectively). The MCA4 epitope in P1 is highlighted using red balls. Values correspond to the distances (in that perspective) between the ends of the C-domains of the two P1 subunits in the Nap complex. Besides the linear displacements the C-domain experiences a hinge rotation of about 175º. (PDF) [file ppat.1012973.s011.pdf]

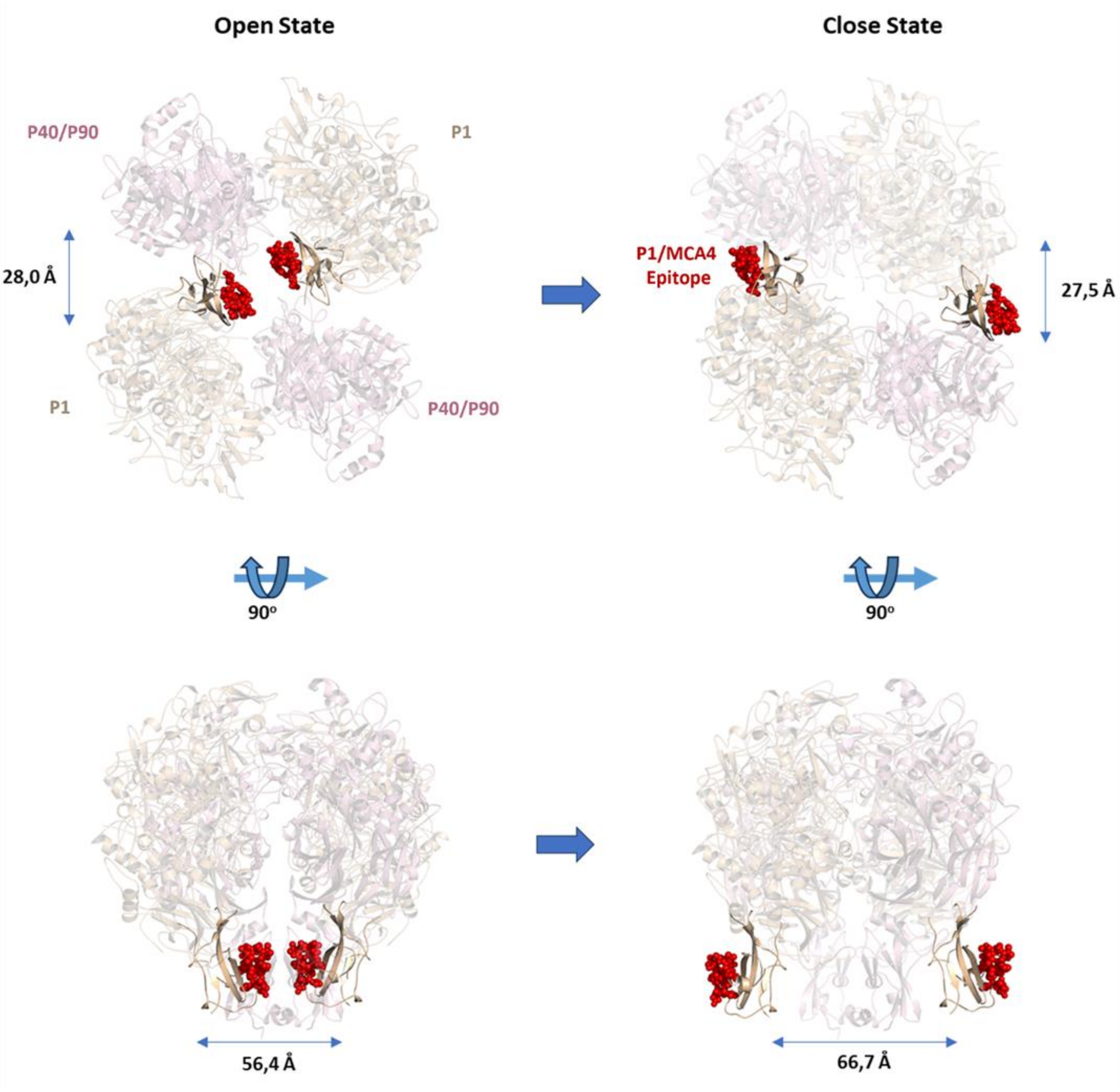

Supplementary Figure 11. C-domain of P1 movements during the "open" to "closed" transition. Top and side views (upper and lower panels, respectively) of the Nap complex ectodomains in the "open" and "closed" conformations (left and right panels, respectively). The MCA4 epitope in P1 is highlighted using red balls. Values correspond to the distances (in that perspective) between the ends of the C-domains of the two P1 subunits in the Nap complex. Besides the linear displacements the C-domain experiences a hinge rotation of about 175º.
