# Supplementary material for: Dynamics of the adhesion complex of the human pathogens Mycoplasma pneumoniae and Mycoplasma genitalium
Source: PLoS Pathog. 2025 Mar 28;21(3):e1012973. doi: 10.1371/journal.ppat.1012973 (PMC11984735; doi:10.1371/journal.ppat.1012973)
Supplement: S1 Table — (PDF) [file ppat.1012973.s012.pdf]

Supplementary Table 1

MCA Monoclonal antibodies generated against the rP1 antigen (A1160-Q1518)

Clone No.	ELISA screening against rP1	Western blot against M129 lysate	Binding to live M129 cells	Inhibition of hemadsorption (HA)	Inhibition of gliding
3	+	+	+	+	+
4	+	+	+	+	+
5	+	+	+	+	+
8	+	+	+	+	+
18	+	+	+	+	+
102	+	-	-	-	-
104	+	+	-	-	-
115	+	+	-	-	-
127	+	+	-	-	-
128	+	+	-	-	-
