# Supplementary material for: Dynamics of the adhesion complex of the human pathogens Mycoplasma pneumoniae and Mycoplasma genitalium
Source: PLoS Pathog. 2025 Mar 28;21(3):e1012973. doi: 10.1371/journal.ppat.1012973 (PMC11984735; doi:10.1371/journal.ppat.1012973)
Supplement: S2 Table — (PDF) [file ppat.1012973.s013.pdf]

**Supplementary Table 2**

**Cryo-EM data collection of the P1-Fab complex and model refinement of the P1-Fab complex**

| **PDB ID** | 8ROR |
|---|---|
| **Data collection** | |
| Microscope | CRYO ARM 300 (JEOL) |
| Voltage (kV) | 300 |
| Detector | K3 direct (Gatan) |
| Data collection software | SerialEM |
| Energy Filter | Slit width of 20 eV |
| Electron dose ($e^-$/ Å$^2$)/Frame | 2.0 |
| Pixel size (Å) | 0.49 |
| Defocus range (µm) | -0.5 to -1.5 |
| Frames | 50 |
| Movie number | 27122 |
| **Data processing** | |
| Processing software | RELION-3.1 |
| Number of extracted particles | 4312408 |
| EMDB code | EMD-19402 |
| Map resolution (Å) at: FSC=0.143 (unmasked/masked) | 2.39 |
| **Refinement** | |
| Software | PHENIX |
| Models used (PDB code) | 6RC9 |
| Atoms (Non-H) | 14210 |
| Protein residues | 1789 |
| Ligands | - |
| Waters | 269 |
| Bonds (r.m.s.d) | |
| Length (Å) | 0.004 |
| Angles (o) | 0.659 |
| B-factor (Å$^2$) | 83.53 |
| MolProbity score | 2.72 |
| Clashcore | 27.36 |
| Rotamer outliers (%) | 3.79 |
| Ramachandran plot | |
| Favored (%) | 94.82 |
| Allowed (%) | 5.07 |
| Disallowed (%) | 0.11 |
| CC (volume) | 0.83 |
| CC (mask) | 0.83 |
