# Supplementary material for: Dynamics of the adhesion complex of the human pathogens Mycoplasma pneumoniae and Mycoplasma genitalium
Source: PLoS Pathog. 2025 Mar 28;21(3):e1012973. doi: 10.1371/journal.ppat.1012973 (PMC11984735; doi:10.1371/journal.ppat.1012973)
Supplement: S3 Table — (PDF) [file ppat.1012973.s014.pdf]

Supplementary Table 3
Monoclonal/polyclonal antibodies inhibition assays (extended data)

PCA	MCN replicate	Before AS Total	Before AS Non-Motile	Before AS Motile	%Motile Cells	After AS Total	After AS Non-Motile	After AS Motile	%Motile Cells	RT_{50} (min)	Detached cells	% Detached cells
**P1**	**1**	436	44	392	89,9	111	105	6	5,4	6,95	325	74,5
	**2**	225	22	203	90,2	73	73	0	0,0	3,99	152	67,6
	**3**	135	16	119	88,1	34	34	0	0,0	3,00	101	74,8
				**Mean**	**89,4**			**Mean**	**1,8**	**4,65**		**72,3**
				**SE**	**0,6**			**SE**	**1,8**	**1,19**		**2,4**
**P40/P90**	**1**	464	40	424	91,4	133	40	93	69,9	NA	331	71,3
	**2**	110	18	92	83,6	89	19	70	78,7	NA	21	19,1
				**Mean**	**87,5**			**Mean**	**74,3**			**45,2**
				**SE**	**3,9**			**SE**	**4,4**			**26,1**
**P1N-ter**	1	575	25	550	95,7	231	30	201	87,0	NA	344	59,8
	2	581	26	555	95,5	234	38	196	83,8	NA	347	59,7
				**Mean**	**95,6**			**Mean**	**85,4**			**59,8**
				**SE**	**0,1**			**SE**	**1,6**			**0,1**
**Negative Control**	**1**	242	21	221	91,3	123	21	102	82,9	NA	119	49,2
	**2**	110	10	100	90,9	60	13	47	78,3	NA	50	45,5
	**3**	302	38	264	87,4	121	40	81	66,9	NA	181	59,9
				**Mean**	**89,9**			**Mean**	**76,1**			**51,5**
				**SE**	**1,2**			**SE**	**4,8**			**4,3**

		Before AS				After AS				
**PCA**	**MCN replicate**	**Total**	**Non-Motile**	**Motile**	**%Motility**	**Total**	**Non-Motile**	**Motile**	**%Motility**	**RT_{50} (min)**
**P1**	**1**	175	5	170	97,1	172	172	0	0	3,40
	**2**	162	6	156	96,3	168	168	0	0	3,04
	**3**	415	11	404	97,3	408	338	70	17,2	5,38
	**4**	360	8	352	97,8	372	361	11	3,0	4,61
	**5**	527	20	507	96,2	424	424	0	0	4,08
	**6**	692	40	652	94,2	692	692	0	0	3,79
				**Mean**	**96,5**			**Mean**	**3,4**	**4,05**
				**SE**	**0,5**			**SE**	**2,8**	**0,35**
**P40/P90**	**1**	181	7	174	96,1	149	7	142	95,3	NA
	**2**	313	9	304	97,1	340	13	327	96,2	NA
	**3**	216	9	207	95,8	183	8	175	95,6	NA
				**Mean**	**96,4**			**Mean**	**95,7**	
				**SE**	**0,4**			**SE**	**0,3**	
**P1N-ter**	**1**	524	52	472	90,1	441	55	386	87,5	NA
	**2**	424	87	337	79,5	453	87	366	80,8	NA
	**3**	747	49	698	93,4	747	54	693	92,8	NA
	**4**	588	43	545	92,7	588	49	539	91,7	NA
				**Mean**	**88,9**			**Mean**	**88,2**	
				**SE**	**3,2**			**SE**	**2,7**	
**Negative Control**	**1**	117	6	111	94,9	114	6	108	94,7	NA
	**2**	230	8	222	96,5	257	12	245	95,3	NA
	**3**	176	6	170	96,6	177	6	171	96,6	NA
	**4**	124	4	120	96,8	125	4	121	96,8	NA
	**5**	417	18	399	95,7	418	16	402	96,2	NA
				**Mean**	**96,3**			**Mean**	**96,5**	
				**SE**	**0,3**			**SE**	**0,1**	

		Before AS				After AS				
**MCA**	**MCN replicate**	**Total**	**Non-Motile**	**Motile**	**%Motility**	**Total**	**Non-Motile**	**Motile**	**%Motility**	**RT_{50} (min)**
**P1-MCA4**	**1**	349	5	344	98,6	349	349	0	0	2,63
	**2**	184	6	178	96,7	168	168	0	0	2,18
	**3**	142	5	137	96,5	142	142	0	0	3,09
				**Mean**	**97,3**			**Mean**	**0**	**2,6**
				**SE**	**0,7**			**SE**	**0**	**0,26**
