# Supplementary material for: Dynamics of the adhesion complex of the human pathogens Mycoplasma pneumoniae and Mycoplasma genitalium
Source: PLoS Pathog. 2025 Mar 28;21(3):e1012973. doi: 10.1371/journal.ppat.1012973 (PMC11984735; doi:10.1371/journal.ppat.1012973)
Supplement: S4 Table — (PDF) [file ppat.1012973.s015.pdf]

Supplementary Table 4
Engelman motif mutants obtained

Strain	Synonyms	Mutation Description
**G37**	*Mge*-WT	-
**G37ΔAdh**	G37ΔMG_191-ΔMG_192	Deletion of the MG_191 and MG_192 genes by allelic exchange.
**G37ΔAdh::COM**	G37ΔMG_191-ΔMG_192::MG_191-MG_192	Re-introduction, by transposon insertion, of a MG_191 and MG_192 wild-type alleles in a G37ΔAdh mutant. It is resistant to puromycin.
**G37ΔAdh::COM (MutE1)**	G37ΔAdh::COM (E1)	Re-introduction of a MG_191 and MG_192 alleles bearing **P140: G1372F-G1376F** substitutions, in a G37ΔAdh mutant. It is resistant to puromycin.
**G37ΔAdh::COM (MutE2; MutE3)**	G37ΔAdh::COM (E2; E3)	Re-introduction of a MG_191 and MG_192 alleles bearing **P110a: G947F-G951F** and **P110b: G960F-G964F** substitutions, in a G37ΔAdh mutant. It is resistant to puromycin.
**G37ΔAdh::COM (MutE1; MutE2; MutE3)**	G37ΔAdh::COM (E1; E2; E3)	Re-introduction of a MG_191 and MG_192 alleles bearing **P140: G1372F-G1376F**, **P110a: G947F-G951F** and **P110b: G960F-G964F** substitutions, in a G37ΔAdh mutant. It is resistant to puromycin.
**G37ΔAdh::COM (MutE2)**	G37ΔAdh::COM (E2)	Re-introduction of a MG_191 and MG_192 alleles bearing **P110a: G947F-G951F** substitutions, in a G37ΔAdh mutant. It is resistant to puromycin.

Strain	Synonyms	Mutation Description
**G37ΔAdh::COM (MutE3)**	G37ΔAdh::COM (E3)	Re-introduction of a MG_191 and MG_192 alleles bearing **P110b: G960F-G964F** substitutions, in a G37ΔAdh mutant. It is resistant to puromycin.
**G37ΔAdh::COM (MutE1; MutE2)**	G37ΔAdh::COM (E1; E2)	Re-introduction of a MG_191 and MG_192 alleles bearing **P140: G1372F-G1376F** and **P110a: G947F-G951F** substitutions, in a G37ΔAdh mutant. It is resistant to puromycin.
**G37ΔAdh-COM (MutE1; MutE3)**	G37ΔAdh-COM (E1; E3)	Re-introduction of a MG_191 and MG_192 alleles bearing **P140: G1372F-G1376F** and **P110b: G960F-G964F** substitutions, in a G37ΔAdh mutant. It is resistant to puromycin.
