# Supplementary material for: Dynamics of the adhesion complex of the human pathogens Mycoplasma pneumoniae and Mycoplasma genitalium
Source: PLoS Pathog. 2025 Mar 28;21(3):e1012973. doi: 10.1371/journal.ppat.1012973 (PMC11984735; doi:10.1371/journal.ppat.1012973)
Supplement: S5 Table — (PDF) [file ppat.1012973.s016.pdf]

Supplementary Table 5

Strain	Individuals Cells	Cells with mTOs	Cells with no TOs (spheres)	Cells with mTOs (%)
**E1**	469	28	2	6,0
**E1; E2**	638	51	14	8,0
**E1; E3**	905	203	15	22,4
**E2; E3**	117	88	4	75,2
**E1; E2; E3**	248	142	7	57,3
**WT**	539	33	0	6,1

Quantification of the frequency of cells with multiple terminal organelles (mTO) and cells with no terminal organelle for each strain.
