# Supplementary material for: Dynamics of the adhesion complex of the human pathogens Mycoplasma pneumoniae and Mycoplasma genitalium
Source: PLoS Pathog. 2025 Mar 28;21(3):e1012973. doi: 10.1371/journal.ppat.1012973 (PMC11984735; doi:10.1371/journal.ppat.1012973)
Supplement: S6 Table — (PDF) [file ppat.1012973.s017.pdf]

Supplementary Table 6
Primers used for P1 and P40/P90 protein constructs expression

**Name**	**Sequence (5'→3')**
P1F	AGGAGATATACCATGACCGTGGTTGGTCACTTTACC
P1R	GTGATGGTGATGTTTATCCGGCCACTGGTTGAACGG
P1F_2	AGGCCATGGCGGCCTTTCGTGGCAGTTG
P1R_2	GTGCTCGAGTCATAAATACTAAGCGGGTT
P1Ct1400_F	AGGAGATATACCATGGCGGATACCGGTCCGCAG
P1Ct1376_F	AGGAGATATACCATGAAAATGAACGATGACGTTG
P40P90_F	AGGAGATATACCATGAGCCTGGCGAACACCTATCTGCTG
P40P90_R	GTGATGTGTATGTTTGCTCGGCACGCGCCGCAAAACC
