# Supplementary material for: Dynamics of the adhesion complex of the human pathogens Mycoplasma pneumoniae and Mycoplasma genitalium
Source: PLoS Pathog. 2025 Mar 28;21(3):e1012973. doi: 10.1371/journal.ppat.1012973 (PMC11984735; doi:10.1371/journal.ppat.1012973)
Supplement: S7 Table — (PDF) [file ppat.1012973.s018.pdf]

Supplementary Table 7

Plasmids used for expression of P1 protein fragments for epitope mapping of P1/MCA4

No.	Plasmids	Primers used for plasmid construction*		Plasmid size	Recombinant P1 fragment	M.W. (kDa) **	Bind to P1/MC4
1	pP1-8	5'-AGGCCATGGCGGCCTTTCGTGGCAGTTG -3'	5'-GTGCTCGAGTCATAAATACTAAGCGGGTT -3'	6789	A1160 - Q1518	43,4	+
2	pP1-8d-N1	5'-TTATTGATTAGTATTTATGACTCGAGCAC -3'	5'-AATACTAATCAATAAACAGCGGTATGT -3'	6246	A1160 - D1446	35,4	+
3	pP1-8d-N2	5'-CGCTGTTTTAGTATTTATGACTCGAGCAC -3'	5'-AATACTAAAACAGCGGTATGTACTGGT -3'	6240	A1160 - F1444	35,2	+
4	pP1-8d-N3	5'-ACATACCGTAGTATTTATGACTCGAGCAC -3'	5'-AATACTACGGTATGTACTGGTCATACA -3'	6234	A1160 - P1442	34,9	+
5	pP1-8d-N4	5'-ACCAGTACTAGTATTTATGACTCGAGCAC -3'	5'-AATACTAGTACTGGTCATACACCAACA -3'	6228	A1160 - Y1440	34,7	+
6	pP1-8d-N5	5'-TGTATGACTAGTATTTATGACTCGAGCAC -3'	5'-AATACTAGTCATACACCAACATAGTTA -3'	6222	A1160 - D1438	34,4	+
7	pP1-8d-N6	5'-TGGTGTATTAGTATTTATGACTCGAGCAC -3'	5'-AATACTAATACACCAACATAGTTACCG -3'	6219	A1160 - Y1437	34,3	-
8	pP1-8d-N7	5'-TGTTGGTGTAGTATTTATGACTCGAGCAC -3'	5'-AATACTACACCAACATAGTTACCGGAT -3'	6216	A1160 - V1436	34,1	-
9	pP1-8d-N8	5'-TAACTATGTAGTATTTATGACTCGAGCAC -3'	5'-AATACTACATAGTTACCGGATCAAACA -3'	6210	A1160 - M1434	33,9	-

No.	Plasmids	Primers used for plasmid construction*		Plasmid size	Recombinant P1 fragment	M.W. (kDa) **	Bind to P1/MC4
10	pP1-8d-N9	5'-ATCCGGTATAGTATTTATGACTCGAGCAC -3'	5'-AATACTATACCGGATCAAACAGATCGG -3'	6204	A1160 – V1432	33,7	-
11	pP1-8d-C1	5'-AGGCCATGACTATGTTGGTGTATGACCA -3'	5'-ACATAGTCATGGCCTTGTCGTCGTC -3'	5970	T1433 - Q1518	14,7	-
12	pP1-8d-C2	5'-AGGCCATGCCGGTAACTATGTTGGTGTA -3'	5'-TTACCGGCATGGCCTTGTCGTCGTC -3'	5976	P1431 - Q1518	14,9	-
13	pP1-8d-C3	5'-AGGCCATGTTTGATCCGGTAACTATGTT -3'	5'-GATCAAACATGGCCTTGTCGTCGTC -3'	5982	F1429 - Q1518	15,2	-
14	pP1-8d-C4	5'-AGGCCATGGATCTGTTTGATCCGGTAAC -3'	5'-ACAGATCCATGGCCTTGTCGTCGTC -3'	5988	D1427 - Q1518	15,4	-
15	pP1-8d-C5	5'-AGGCCATGACCGATCTGTTTGATCCGGT -3'	5'-GATCGGTCATGGCCTTGTCGTCGTC -3'	5991	T1426 - Q1518	15,5	+
16	pP1-8d-C6	5'-AGGCCATGGTCACCGATCTGTTTGATCC -3'	5'-CGGTGACCATGGCCTTGTCGTCGTC -3'	5994	V1425 - Q1518	15,6	+
17	pP1-8d-C7	5'-AGGCCATGGCTGACACTGGTCCACAA -3'	5'-TGTCAGCCATGGCCTTGTCGTCGTC -3'	6069	A1400 – Q1518	18,4	+
18	pP1-8d-C8	5'-AGGCCATGAAGATGAATGACGATGTT -3'	5'-TCATCTTCATGGCCTTGTCGTCGTC -3'	6141	K1376 – Q1518	21	+

The pP1-8 plasmid was constructed by inserting a PCR amplified fragment from P1 coding gene into the NcoI and XhoI sites of pET-30c(+) expression vector.

The remaining plasmids were constructed by deletion of the pP1-8 using specific primer set and PrimeSTAR Mutagenesis Basal kit.

* NcoI and XhoI sites of the primers used for pP1-8 plasmid construction are underlined.

** Molecular weight of recombinant P1 fragments including His- and S-tag sequences (45 aa: 5 kDa) from the pET-30c(+) expression vector.
