# Supplementary material for: Dynamics of the adhesion complex of the human pathogens Mycoplasma pneumoniae and Mycoplasma genitalium
Source: PLoS Pathog. 2025 Mar 28;21(3):e1012973. doi: 10.1371/journal.ppat.1012973 (PMC11984735; doi:10.1371/journal.ppat.1012973)
Supplement: S8 Table — (PDF) [file ppat.1012973.s019.pdf]

Supplementary Table 8
Primers used in the generation of M. genitalium adhesins constructs

Primer Name	Sequence 5' -3'	Use
	**Double adhesins null mutant**	
MgParUp-F	ATCATTACCATTATCAATG	Build pBEΔAdh
MgParUp-R	GGATCCATGCACCTCTCGAGACAAACTTAATTATAAACAAT	
MgParDw-F	CTCGAGAGGTGCATGGATCCTAGTTTTTAACCTTTCAATAAC	
MgParDw-R	CTACTATTGCTAGGTTTCAC	
Lox71p438Fwd-XhoI	CTC GAG TAC CGT TCG TAT AAT GTA TGC TAT ACG AAG TTA TTA GTA TTT AGA ATT AAT AAA GT	
CatLox66Rev-BamHI	GGA TCC TAC CGT TCG TAT AGC ATA CAT TAT ACG AAG TTA TTT ACG CCC CGC CCT GCC ACT	
	**Complementation of the null mutant**	
COMmg191-F-ApaI	AGTGGGCCCACTAACAAAAAACAAATTAGTGATG	Build pMTnPacCOM (MG_191/MG_192)
COMmg191/192-R-SalI	AGTGTCGACATCCACTCTCTAAATTGCAAGTTTAG	
	**Engelman Motif mutants**	
EngMotifP140-F	TTTTTAACGATTTTTATTCCAATGCACAGAAAC	Build pMTnPacCOM:MG_191/MG_192 with the different Engelman Motif substitutions
EngMotifP140-R	*CAAAGTAACACTAAGGATAATC	
EngMotifP110-F	TTTTCTTCAGTTTTTATCTTGTTTATCTTGTTAGTCTTATTTCTTGGGATTTTTATCCCAATGTACAGGGTAAGA	
EngMotifP110a-F	TTTTCTTCAGTTTTTATCTTGTTTATCTTGTTAGTC	
EngMotifP110-R	*TACTGATACAGGGATCACCCA	
EngMotifP110b-F	TTTCTTGGGATTTTTATCCCAATGTACAGGGTAAGA	
EngMotifP110b-R	*TAAGACTAACAAGATAAACAAG	

Primer Name	Sequence 5' -3'	Use
**Sequencing primers**		
Pac-Up	GTAGCTAATCTAACAGTAGG	To sequence DNA inserts cloned in a miniPac Tnp
Pac-Dw	GTCCTAGAACTTGGTGTATG	To determine miniTnPac Tnp insertion point
COMmg191-R-SalI	AGTGTCGACttattgttttactggaggttt	To sequence DNA inserts cloned in a miniPac Tnp
Fup17	GTAAAACGACGGCCAGT	Universal Forward primer
Rup17	GGAAACAGCTATGACCATG	Universal Reverse primer
Tnp3	CATGATGAATGGATTTATTC	To sequence DNA inserts cloned in a miniPac Tnp
RTPCR192-F	GTTGATACACTCACAACTG	To sequence DNA inserts cloned in a miniPac Tnp
RTPCR192-R	CTAACTTTTGGTTTCTTCTGAC	To sequence DNA inserts cloned in a miniPac Tnp
RTPCRmg191-F	CTGGAGAGAACCCAGGATCA	To sequence DNA inserts cloned in a miniPac Tnp
SEQmg191a-F	GGTTAGTTTCTATGATGCAC	To sequence DNA inserts cloned in a miniPac Tnp
SEQmg191b-F	GATACAGCTACTGTACCTAG	To sequence DNA inserts cloned in a miniPac Tnp
SEQmg191c-F	GTTCTACCTTCGATCAGTTC	To sequence DNA inserts cloned in a miniPac Tnp
SEQmg191d-F	GCATTACTCCATACCTATGG	To sequence DNA inserts cloned in a miniPac Tnp
SEQmg191e-F	ATTAACACCATCACCACTAC	To sequence DNA inserts cloned in a miniPac Tnp
**Oligonucleotides used in mutant screening**		
SCRMgPaFw	GTCTGTTTGCCATCTATGACA	To screen for MG_191/MG_192 null mutants
SCRMgPaRev	GAGCCACCTGAAGTGACTT	
