# Supplementary material for: Dynamics of the adhesion complex of the human pathogens Mycoplasma pneumoniae and Mycoplasma genitalium
Source: PLoS Pathog. 2025 Mar 28;21(3):e1012973. doi: 10.1371/journal.ppat.1012973 (PMC11984735; doi:10.1371/journal.ppat.1012973)
Supplement: S9 Table — (PDF) [file ppat.1012973.s020.pdf]

Supplementary Table 9
Antibodies used in this work

Antibody	Host	Dilution	Conjugated enzyme	Source
**Anti-mouse IgG (H+L) Alexa Fluor 555**	Goat	1:250		Invitrogen
**Polyclonal anti-Mouse IgG (H+L)**	Goat	1:3000	HRP	Bio-Rad
**Polyclonal anti-MG_191/MG_192 (Nap)**	Mouse	1:1000	-	This work
**Polyclonal anti-MPN141 (P1)**	Mouse	1:10/1:1000	-	This work
**Polyclonal anti-MPN142 (P40P90)**	Mouse	1:10/1:1000	-	This work
**Polyclonal anti-MPN141 N-domain**	Mouse	1:10/1:1000	-	This work
**Monoclonal anti-MPN141 (P1/MCA4)**	Mouse?	1:100/1:3000	-	Seto *et al.* 2005 (unpublished). This work
